# A Mixture of Valine and Isoleucine Restores the Growth of Protein-Restricted Pigs Likely through Improved Gut Development, Hepatic IGF-1 Pathway, and Plasma Metabolomic Profile

**DOI:** 10.3390/ijms23063300

**Published:** 2022-03-18

**Authors:** Mohammad Habibi, Parniyan Goodarzi, Cedrick Ndhumba Shili, Julia Sutton, Caitlyn Marie Wileman, Dohyung Markus Kim, Dingbo Lin, Adel Pezeshki

**Affiliations:** 1 Department of Animal and Food Sciences, Oklahoma State University, Stillwater, OK 74078, USA; mohammad.habibi@okstate.edu (M.H.); parniyan.goodarzi@okstate.edu (P.G.); cedrick.shili@okstate.edu (C.N.S.); julia.sutton@okstate.edu (J.S.); caitlyn.wileman@okstate.edu (C.M.W.); dohyung.kim@okstate.edu (D.M.K.); 2 Department of Nutritional Sciences, Oklahoma State University, Stillwater, OK 74078, USA; dingbo.lin@okstate.edu

**Keywords:** low protein, isoleucine, valine, growth, gut development, insulin-like growth factor 1, metabolomics, pig

## Abstract

Valine (Val) alone or in combination with isoleucine (Ile) improves the growth under severe protein restriction; however, the underlying mechanisms remain unknown. In this study, we assessed whether Val/Ile-induced growth in protein-restricted pigs is associated with changes in gut development, hepatic insulin-like growth factor 1 (IGF-1) production, and blood metabolomics. Forty piglets were assigned to five dietary groups: positive control (PC) with standard protein content; low protein (LP) with very low protein content; and LP supplemented with Val (LPV), Ile (LPI), and Val and Ile (LPVI). LPVI reversed the negative effects of VLP diets on growth and gut morphology. Both LPV and LPVI restored the reduced transcript of IGF-1 while decreasing the transcript of insulin-like growth factor binding protein 1 (IGFBP1) in the liver. LPV and LPVI recovered the reduced plasma Val, glycine, and leucine concentrations, which were positively correlated with improved gut morphology and the hepatic IGF-1 gene expression and negatively correlated with hepatic IGFBP1 mRNA abundance. In conclusion, supplementation with a combination of Val and Ile into the VLP diets restored the decreased growth performance of pigs fed with these diets likely through improved gut development, hepatic IGF-1 expression and bioavailability, and plasma metabolomics profile.

## 1. Introduction

Slightly low-protein (SLP) diets (reduced crude protein (CP) by 4% unit) supplemented with the first four limiting amino acids (LAA), i.e., lysine (Lys), methionine (Met), threonine (Thr), and tryptophan (Trp), have been suggested for reducing nitrogen (N) excretion from swine production [1] without having a negative influence on the growth performance of pigs [2]. While decreasing dietary CP by more than 4% unit may produce a higher reduction in N excretion than SLP diets in pigs [3], very-low-protein (VLP) diets fail to maintain normal growth in pigs even when supplemented with first four LAA [4,5,6,7]. This could be the indication of the importance of next LAA for maintaining the optimal growth of pigs under VLP diets. Further studies are required to identify the next LAA and characterize their optimal combinations for pigs fed with VLP diets.

In an effort to assess the effect of branched-chain amino acids (BCAA), i.e., leucine (Leu), isoleucine (Ile), and valine (Val), on the growth of pigs fed with VLP diets, we reported a partial recovery of growth when VLP diets supplemented with the first four LAA and BCAA at NRC [8] or above NRC levels [4]. However, BCAA together with the first four LAA enhanced the growth performance in weaner pigs fed with SLP diets [9,10]. BCAA are required for several metabolic pathways such as protein synthesis, energy homeostasis, and intestinal development [11,12]. Among BCAA, Leu has been shown to reduce feed intake (FI) [13,14] through the activation of mammalian target of rapamycin complex 1 (mTORC1) pathway in the brain [15]. Likewise, Ile supplementation to VLP diets together with Lys, Thr, Met, and Trp in gilts [16] or with Lys, Thr, and Trp in growing pigs [17] appears to aggravate the reduced FI, weight gain, and feed efficiency and depress the growth rate. However, Val alone or in combination with Ile has shown to improve the growth in pigs fed with VLP [16,17,18,19,20,21] or SLP [22,23,24,25] diets containing adequate amounts of the first four LAA. The mechanisms by which Ile and Val improve the growth during protein restriction remain largely elusive.

Branched-chain amino acids potentially influence the growth of pigs through improving muscle protein synthesis [26], amino acid (AA) utilization [27], FI [4,28,29], intestinal development and immune function [9,10,30], blood metabolites [31] and gut microbiota profile [8], calcium absorption/reabsorption [32], and insulin-like growth factor 1 (IGF-1) signaling [4]. It has been shown that dietary Ile supplementation alone increases the glucose uptake [33], enhances the intramuscular fat content and monounsaturated fatty acid synthesis in muscle [34], and influences the immune function [35] in pigs. Additionally, increasing the dose of dietary Val alone influenced the circulating and tissues AA and metabolites profile [25]. Whether modulation of growth by dietary Val and Ile (individually or as mixture) in pigs fed with VLP diets is related with changes in blood metabolomics, gut development, and hepatic IGF-1 production is yet to be understood. Therefore, we aimed to examine the effect of Val and Ile or a combination of both on the growth performance of pigs fed with VLP diets containing the first four LAA and assess whether that is associated with alterations in blood metabolomics, gut development, and hepatic IGF-1 expression.

## 2. Results

### 2.1. Growth Measurements

Initial body weight (BW) was not different among groups (Table 1). Compared with the positive control (PC, i.e., basal diet with standard protein content), low-protein diet (LP, i.e., basal diet with very low protein content containing LAA) and LPI (i.e., LP containing Ile at levels suggested by National Research Council [NRC]) had a lower final BW, but LPV (i.e., LP containing Val at NRC level) and LPVI (i.e., LP containing both Val and Ile at NRC levels) were not different compared with PC. Both LPV and LPVI had higher final BW than LPI, but only LPVI had higher final BW than LP. Relative to PC, LP, LPV, and LPI reduced the average daily gain (ADG) by 44, 25, and 58%, respectively (Table 1). The ADG of pigs fed with LPVI was not different than PC and LPV pigs and that was 56 and 110% higher than LP and LPI, respectively. LPI had lower ADG when compared to LPV and LP (Table 1). Compared with PC, LPI reduced the average daily feed intake (ADFI) by 39% (Table 1). The ADFI was 29.5 and 72% higher in LPVI than LP and LPI, respectively (Table 1). In comparison with LPV, ADFI was 33% lower in LPI. The ADFI tended to be lower in LPI (24.5%) compared to LP. Average daily protein intake (ADPI) was lower in all groups compared with PC (Table 1). Moreover, LPI had lower ADPI than LPV and LPVI. No differences were found when ADPI of LPV was compared to that of LPVI (*p* > 0.1). LPVI tended to have a higher ADPI than LP. LPI had lower average daily water intake (ADWI) than PC, LPV, and LPVI, but that was not different when compared to LP (Table 1; *p* < 0.01). Relative to PC, pigs fed with LP, LPV, LPI, and LPVI had 31, 17, 34, and 20% lower gain:feed (G:F), respectively (*p* < 0.01). Both LPV and LPVI had higher G:F than LPI. LPV had higher G:F than LP, and LPVI tended to increase the G:F relative to LP. LPV and LPVI had higher gain:protein (G:P) than LP and LPI and they tended to increase the G:P compared to PC (Table 1; *p* < 0.01). No differences in the water:feed (W:F) ratio were detected among treatments (Table 1). Compared with PC, body length was significantly lower in LP, LPV, and LPI; however, it was unchanged in LPVI. LPVI tended to have a higher body length than LPI. Heart girth was significantly lower in LP and LPI than PC, whereas no significant differences were detected for LPV vs. PC and LPVI vs. PC (Table 1). LPVI and LPV had a higher heart girth than LPI. While wither height was reduced in pigs fed with LP (*p* < 0.01) and LPI relative to PC, no differences in wither height were seen when LPV and LPVI were compared to PC (Table 1). LPVI had higher wither height than LPI and LP and LPV had greater wither height than LPI.

Pigs fed with LP diet had lower body weight gain (BWG) than PC on week 1 and weeks 3–5. While no significant difference was detected when the BWG of PC was compared to LPVI, LPI significantly reduced BWG in comparison with PC and LPVI from weeks 1–5 (Supplementary Table S1; *p* < 0.01). Moreover, except weeks 3 and 5, BWG of LPI was significantly lower than LPV (Supplementary Table S1). LPV and LPVI had similar BWG as LP with temporary higher BWG than LP in certain weeks. Relative to PC, LP had a lower mean feed intake (MFI) in the last two weeks of the study. Relative to PC, MFI was decreased in LPI in all weeks; however, it was unchanged in LPV and LPVI (Supplementary Table S1). Moreover, relative to LPV, LPI reduced MFI by 29–35% from week 1 to 5. In comparison with LPI, LPVI increased MFI by 53–87% in weeks 1–5 (Supplementary Table S1). LPV had similar MFI as LP in all 5 weeks, but LPVI increased the MFI on weeks 2 and 5 compared with LP. In comparison to PC, cumulative feed intake (CFI) of LP was 23.8 and 27.4% lower in weeks 4 and 5, respectively (Supplementary Table S1). LPV and LPVI showed a higher CFI relative to LPI during the whole experiment (Supplementary Table S1). Compared with LP, LPVI increased CFI by 42 and 45% in weeks 2 and 5, respectively, but LPV did not change the CFI (Supplementary Table S1). No differences in CFI were detected for LPV vs. LPVI, LPV vs. PC, and LPVI vs. PC. As expected, LP had lower cumulative protein intake (CPI) than PC throughout the study, but LPV and LPVI did not change CPI in weeks 1 and 2, relative to PC (Supplementary Table S1). Furthermore, relative to LP, CPI was significantly greater in LPVI during the weeks 1, 2, and 5, but that did not change for LPV (Supplementary Table S1). Compared with LPI, LPVI had higher CPI from weeks 1 to 5 and LPV had a greater CPI on weeks 1, 2, 4, and 5 (Supplementary Table S1; *p* < 0.01). Relative to PC, LP had a lower G:F during weeks 1 and 4. No differences in G:F were detected for LPV vs. PC, but LPVI had a lower G:F than PC during weeks 1, 2, and 4. No differences in G:F among LP, LPV, LPI, and LPVI were detected. The G:P did not change among groups during the first 3 weeks of the study. In weeks 4 and 5, except a higher G:P for LPV vs. PC, LP and LPI, and LPVI vs. PC, respectively, no other differences in G:P were detected.

**Figure 1:**
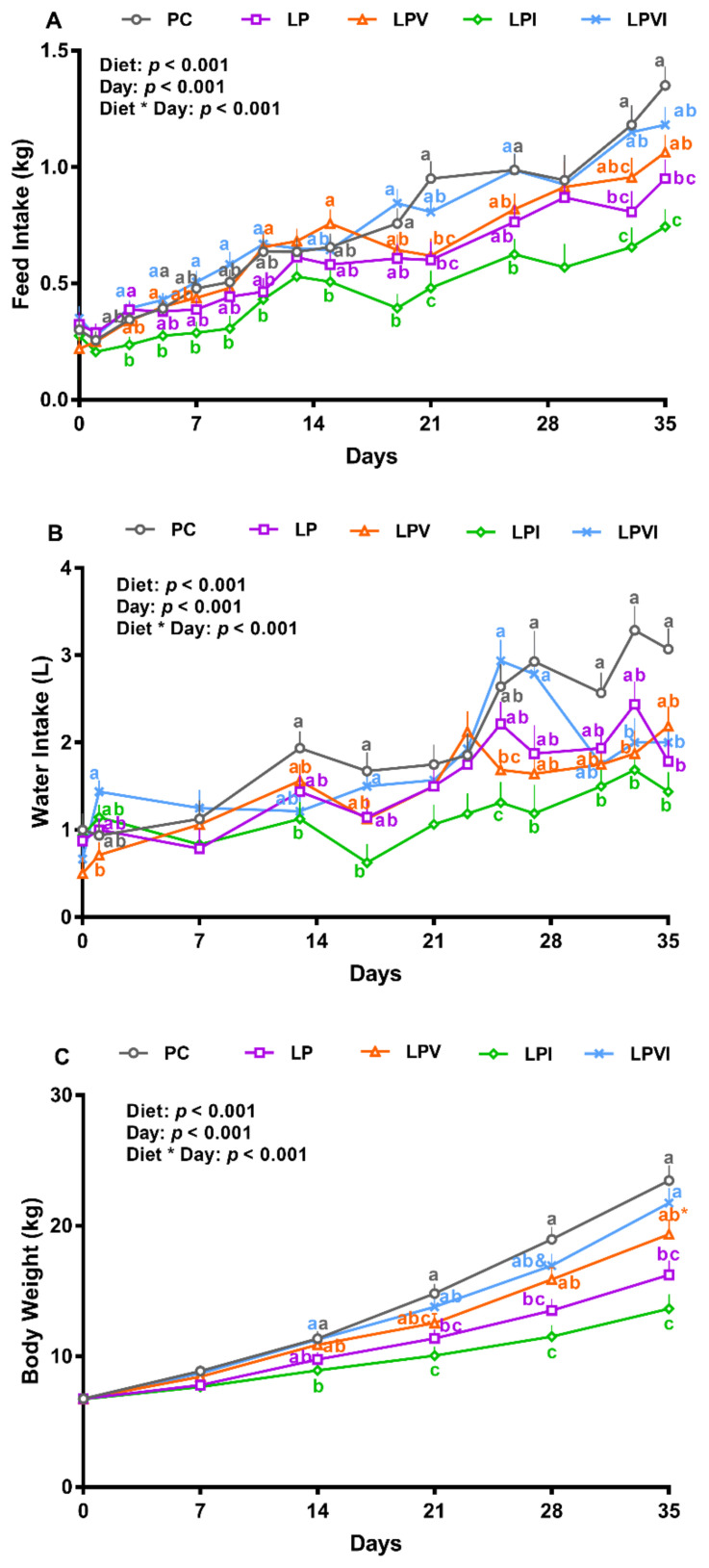
(**A**) Feed intake, (**B**) water intake, and (**C**) body weight of nursery pigs fed with very-low-protein diets supplemented with Ile, Val, or combination of both. PC (positive control): standard-protein diet; LP (negative control): low-protein diet containing limiting amino acids (i.e., Lys, Met, Thr and Trp) at NRC (2012) levels; LPV: LP containing Val at NRC level; LPI: LP containing Ile at NRC level; LPVI: LP containing Val and Ile at NRC levels. The values are means ± standard error of the mean. *n* = 8. ^a,b,c^ Among groups, the means with different superscript letter(s) are different (*p* ≤ 0.05). ^&^ *p* ≤ 0.1 LPVI vs. LP, * *p* ≤ 0.1 LPV vs. PC.

The overall effects of the diet, day, and diet by day on daily FI were significant (Figure 1A, *p* < 0.001). Compared to PC, pigs fed with the LP diet had numerically lower FI for the first three weeks of the experiment; however, they reduced the FI significantly from day 21 onwards (Figure 1A). Supplementation with a combination of Val and Ile into the LP diet (i.e., LPVI) completely recovered the FI to the level of PC throughout the study; however, supplementing Ile into the VLP diet (i.e., LPI) significantly reduced FI from days 3 and 19 onwards, compared with LPVI (by 22–114%) and PC (by 37–50%), respectively (Figure 1A). While Val supplementation into the VLP diet (i.e., LPV) reduced FI by 35% compared with PC on day 21 (*p* < 0.01), no significant differences were found for FI for LPV vs. PC, LP, and LPVI during the experiment (Figure 1A; *p* > 0.1). In comparison with LPI, LPV increased the FI on days 5, 11, 15, and 35 (Figure 1A; *p* < 0.01). Overall, the effects of diet, day, and the interaction of diet by day on daily water intake (WI) were significant (Figure 1B; *p* < 0.01). Relative to PC, WI was 42% lower for LP on day 35 and it was 36–63% lower in LPI following day 13 of the study (Figure 1B; *p* < 0.01). Relative to LPI, LPVI increased the WI by 140, 124, and 134% on days 17, 25, and 27, respectively (Figure 1B). No significant differences were detected when LPV was compared to LP (*p* > 0.1); however, relative to LPV, WI was 101 and 74% higher in LPVI on days 1 and 25, respectively (Figure 1B; *p* < 0.01). The effects of diet, day, and the interaction of diet by day on weekly BW was significant (Figure 1C; *p* < 0.01). In comparison with PC, LP reduced BW by 23, 29, and 31% on days 21, 28, and 35, respectively (Figure 1C; *p* < 0.01). LPI had lower BW from day 14 onwards, when compared with PC or LPVI and from day 28 onwards when compared with LPV (Figure 1C; *p* < 0.01). BW of LPVI tended to be higher than LP on day 28 and it was 34% higher than those fed with LP on day 35 (Figure 1C; *p* < 0.01). No significant differences were found when BW of LPV was compared to that of LP (Figure 1C; *p* > 0.1). The BW of PC, LPV, and LPVI was not significantly different during the experimental period (Figure 1C; *p* > 0.1); however, LPV tended to reduce the BW on day 35 when compared to PC (0.05 < *p* < 0.1).

**Figure 2:**
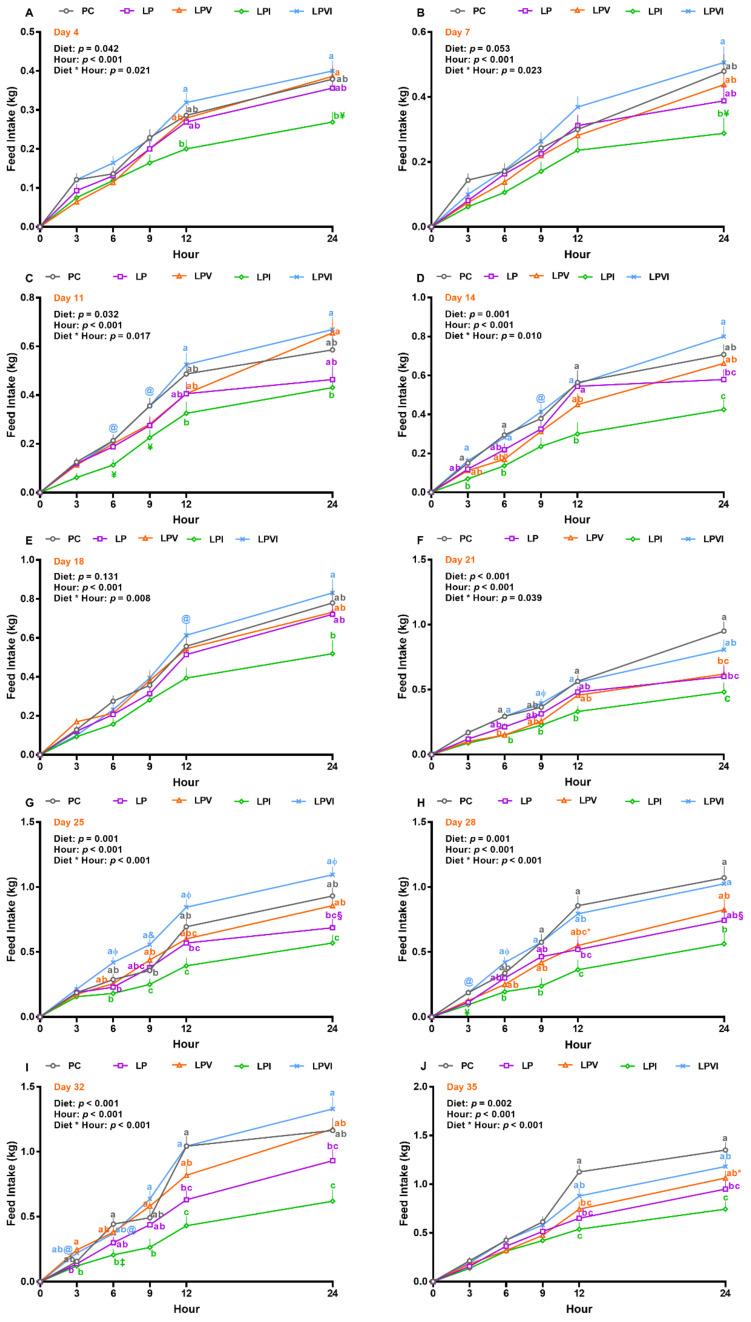
Feed intake at (**A**) Day 4, (**B**) Day 7, (**C**) Day 11 (**D**) Day 14, (**E**) Day 18, (**F**) Day 21, (**G**) Day 25, (**H**) Day 28, (**I**) Day 32, (**J**) Day 35 in nursery pigs fed with very-low-protein diets supplemented with Ile, Val, or combination of both. PC (positive control): standard-protein diet; LP (negative control): low-protein diet containing limiting amino acids (i.e., Lys, Met, Thr and Trp) at NRC (2012) levels; LPV: LP containing Val at NRC level; LPI: LP containing Ile at NRC level; LPVI: LP containing Val and Ile at NRC levels. The values are means ± standard error of the mean. *n* = 8. ^a,b,c^ Among groups, the means with different superscript letter(s) are different (*p* ≤ 0.05). ^§^ *p* ≤ 0.1 LP vs. PC, * *p* ≤ 0.1 LPV vs. PC, ^¥^ *p* ≤ 0.1 LPI vs. PC, ^&^ *p* ≤ 0.1 LPVI vs. LP, ^‡^ *p* ≤ 0.1 LPI vs. LPV, ^ɸ^ *p* ≤ 0.1 LPVI vs. LPV, ^@^ *p* ≤ 0.1 LPVI vs. LPI.

Overall, the effects of diet, hour, and the interaction of diet by hour were significant on hourly FI on days 4, 7, 11, 14, 21, 25, 28, 32, and 35 (Figure 2A–J). On days 4, 11, 14, 21, 25, 28, 32, and 35 FI was lower in LPI than LPVI at hours 12 and 24. Relative to LP and LPI, FI increased in LPVI on days 25, 28, and 32 at hours 12 and 24 (Figure 2G–I). Further, LPVI had a higher FI than LP on day 14 at hour 24 (Figure 2D). While LP had lower FI than PC at hour 24 on day 21 (Figure 2F), hour 12 of days 28 and 32 (Figure 2H,I), and hours 12 and 24 of day 35 (Figure 2J), no differences in FI were detected between PC and LPVI on these days and hours.

Following meal test, no significant differences in FI were detected among dietary treatments (*p* = 0.112). The FI for PC, LP, LPV, LPI, and LPVI was 0.27, 0.26, 0.29, 0.19, and 0.32 kg, respectively.

### 2.2. Thermal Radiation

**Figure 3:**
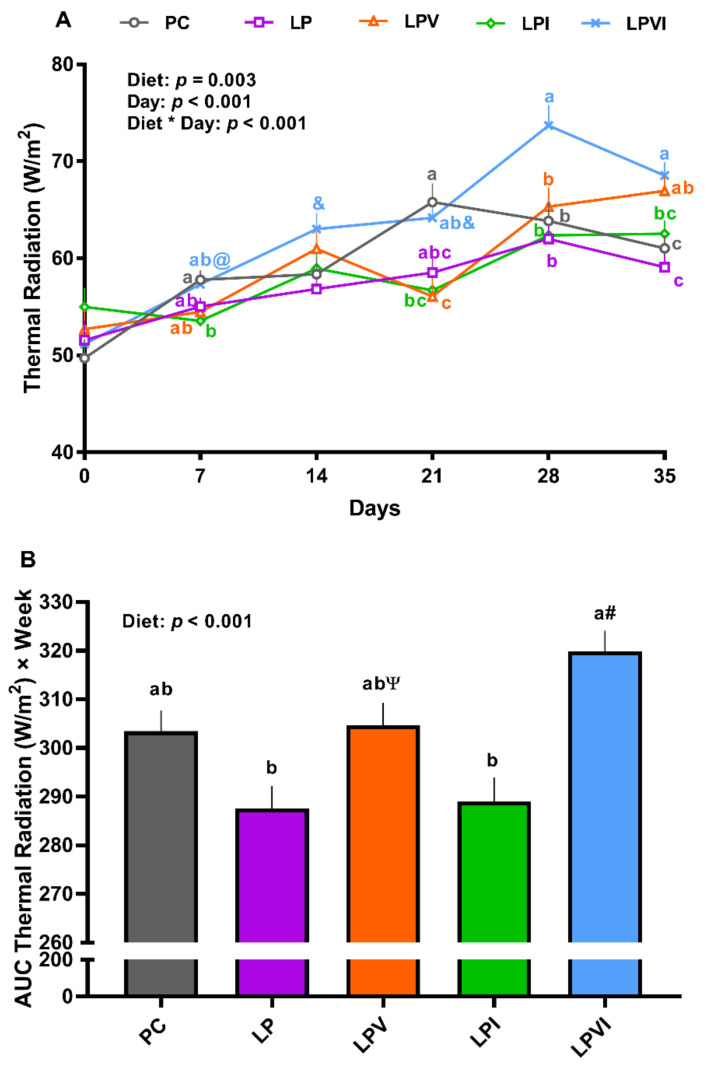
(**A**) Thermal radiation and (**B**) area under the curve (AUC) thermal radiation of nursery pigs fed with very-low-protein diets supplemented with Ile, Val, or combination of both. PC (positive control): standard-protein diet; LP (negative control): low-protein diet containing limiting amino acids (i.e., Lys, Met, Thr and Trp) at NRC (2012) levels; LPV: LP containing Val at NRC level; LPI: LP containing Ile at NRC level; LPVI: LP containing Val and Ile at NRC levels. The values are means ± standard error of the mean. *n* = 8. ^a,b,c^ Among groups, the means with different superscript letter(s) are different (*p* ≤ 0.05). ^#^ *p* ≤ 0.1 LPVI vs. PC, ^Ψ^ *p* ≤ 0.1 LPV vs. LP, ^&^ *p* ≤ 0.1 LPVI vs. LP, ^@^ *p* ≤ 0.1 LPVI vs. LPI.

Overall, the effects of diet, day, and the interaction of diet by day on thermal radiation were significant (Figure 3A; *p* < 0.01). No significant differences were detected when thermal radiation of PC and LP were compared (Figure 3A). Relative to LPI, LPVI tended to increase the thermal radiation on day 7 and significantly increased the thermal radiation on day 28 and 35 (Figure 3A; *p* < 0.01). Compared to LP, LPVI tended to increase the thermal radiation on days 14 and 21 and increased that significantly on days 28 and 35 (Figure 3A). LPVI also increased the thermal radiation on days 28 and 35 relative to PC. LPV had higher thermal radiation on day 35 when compared with LP. The effect of diet on the area under the curve (AUC) of thermal radiation was significant (Figure 3B; *p* < 0.01). Relative to LP and LPI, the AUC thermal radiation was significantly higher in LPVI (Figure 3B; *p* < 0.01). Furthermore, the AUC thermal radiation tended to increase in LPVI in comparison with PC. LPV tended to have a higher AUC thermal radiation than LP (Figure 3B).

### 2.3. Intestinal Histomorphology

**Figure 4:**
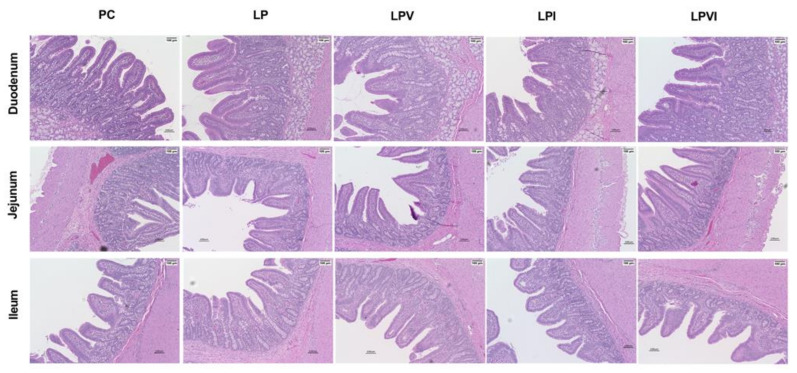
Representative intestinal hematoxylin and eosin-stained sections (×10 magnification) micrographs of duodenum, jejunum, and ileum in pigs fed with very-low-protein diets supplemented with Ile, Val, or combination of both. PC (positive control): standard-protein diet; LP (negative control): low-protein diet containing limiting amino acids (i.e., Lys, Met, Thr and Trp) at NRC (2012) levels; LPV: LP containing Val at NRC level; LPI: LP containing Ile at NRC level; LPVI: LP containing Val and Ile at NRC levels. *n* = 8.

Pigs fed with LP had lower duodenal villus width and crypt depth and higher villus height:crypt depth than those in PC, but no differences in villus height, crypt width, and muscular thickness were seen (Table 2; Figure 4). Except a reduction in crypt depth for LPVI, no changes in duodenal villus height, villus width, crypt width, muscular thickness, and villus height:crypt depth were seen in comparison to PC. Relative to LP, LPVI had a higher duodenal crypt depth and LPV tended to have a higher muscle thickness. Duodenal villus width and crypt depth were decreased in LPI and villus height:crypt depth was increased in LPV relative to PC (Table 2; Figure 4). Duodenal muscular thickness was higher in LPI than LP (Table 2; Figure 4). In jejunum, LP had a lower crypt depth than PC. LPVI tended to have a lower jejunal crypt depth than PC, but no differences were seen relative to LP. LPV and LPI had a lower crypt depth than PC with no differences in comparison with LP. Moreover, LPI reduced the villus height in jejunum compared with LPVI (Table 2; Figure 4). In ileum, LP had a lower villus height and villus width than PC (Table 2; Figure 4). LPVI and LPV had similar ileal histomorphology measures as PC and LPVI had a higher villus height than LP. LPI had a lower ileal villus height, villus width, and crypt width than PC and had a lower villus height than LPV and LPVI. Ileal villus height:crypt depth was higher in LPVI compared with LPI.

### 2.4. The mRNA Abundance of Key Regulatory Genes of Intestinal Development in Duodenum

**Figure 5:**
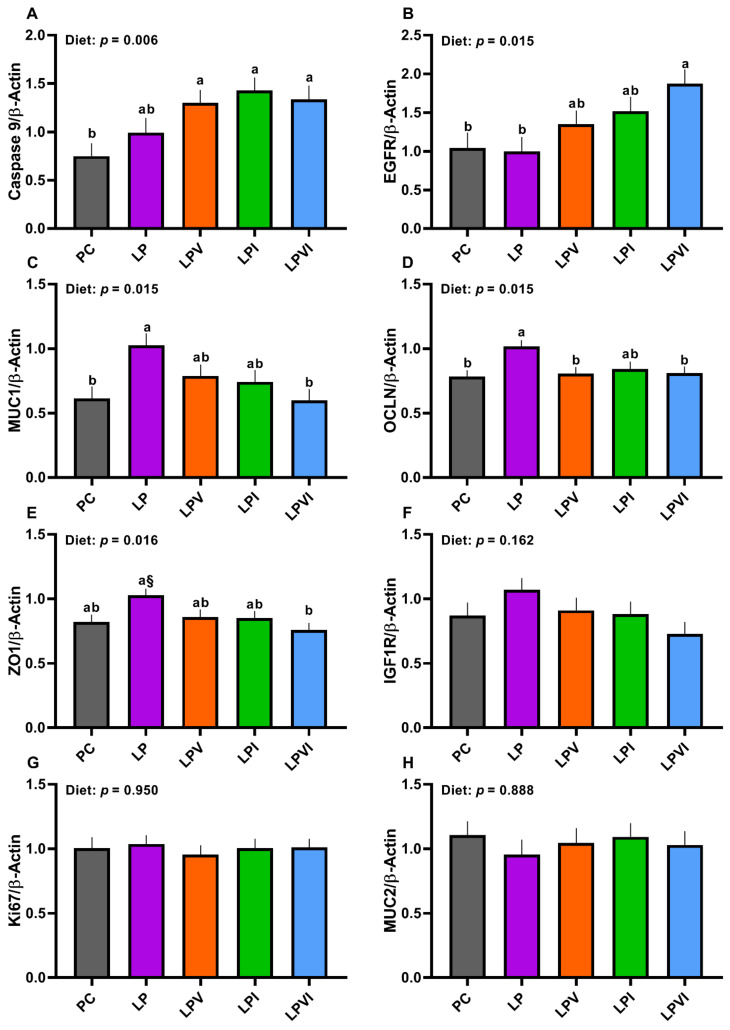
Duodenal mRNA abundance of (**A**) *caspase-9*, (**B**) epidermal growth factor receptor (*EGFR*), (**C**) mucin 1 (*MUC1*), (**D**) occludin (*OCLN*), (**E**) zonula occludens 1 (*ZO1*), (**F**) insulin-like growth factor-1 receptor (*IGF1R*), (**G**) *Ki-67*, and (**H**) mucin 2 (*MUC2*), in nursery pigs fed with very-low-protein diets supplemented with Ile, Val, or combination of both. PC (positive control): standard-protein diet; LP (negative control): low-protein diet containing limiting amino acids (i.e., Lys, Met, Thr and Trp) at NRC (2012) levels; LPV: LP containing Val at NRC level; LPI: LP containing Ile at NRC level; LPVI: LP containing Val and Ile at NRC levels. The values are means ± standard error of the mean. *n* = 8. ^a,b^ Among groups, the means with different superscript letter(s) are different (*p* ≤ 0.05). ^§^ *p* ≤ 0.1 LP vs. PC.

Overall, the effect of diet on the gene expression of duodenal *Caspase 9*, epidermal growth factor receptor (*EGFR*), mucin 1 (*MUC1*), occludin (*OCLN*), and zonula occludens 1 (*ZO1*) was significant (Figure 5A–E). The mRNA abundance of *Caspase 9* was not different between PC and LP diets, but LPVI, LPV, and LPI increased that relative to PC (Figure 5A; *p* < 0.01). The gene expression of *EGFR* was not different between PC and LP, but the gene expression of *EGFR* was greater in LPVI than PC and LP (Figure 5B; *p* = 0.015). In comparison with PC, the gene expression of duodenal *MUC1* was increased in LP (Figure 5C; *p* = 0.015). However, the mRNA abundance of *MUC1* was not different for LPVI, LPV and LPI vs. PC and that was lower for LPVI than LP. Compared with PC, duodenal transcript of *OCLN* was higher in LP (Figure 5D). However, the transcript of *OCLN* was not different for LPVI, LPV, and LPI vs. PC and that was lower for LPV and LPVI than LP. Relative to PC, the mRNA abundance of *ZO1* tended to be higher in LP (Figure 5E). The mRNA abundance of *ZO1* was not different for LPVI, LPV, and LPI vs. PC and that was lower for LPVI than LP. No differences among treatments were detected for the mRNA abundance of duodenal insulin-like growth factor-1 receptor (*IGF1R*) (Figure 5F), *Ki67* (Figure 5G), and mucin 2 (*MUC2*) (Figure 5H).

### 2.5. The mRNA Abundance of Key Molecules of IGF-1 Signaling Pathway in the Liver

**Figure 6:**
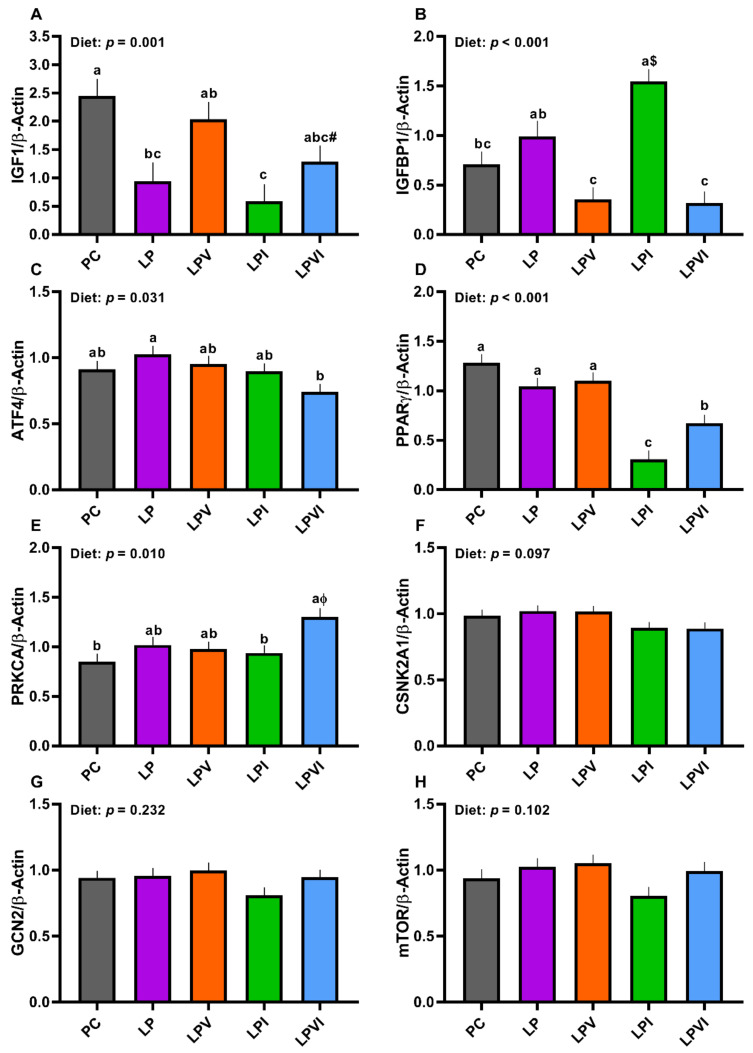
Hepatic mRNA abundance of (**A**) insulin-like growth factor-1 (*IGF1*), (**B**) insulin-like growth factor binding protein 1 (*IGFBP1*), (**C**) activating transcription factor 4 (*ATF4*), (**D**) peroxisome proliferator-activated receptor gamma (*PPARγ*), (**E**) protein kinase C alpha (*PRKCA*), (**F**) casein kinase 2 alpha 1 (*CSNK2A1*), (**G**) general control non-derepressible-2 (*GCN2*), and (**H**) mechanistic target of rapamycin (*mTOR*), in nursery pigs fed with very-low-protein diets supplemented with Ile, Val, or combination of both. PC (positive control): standard-protein diet; LP (negative control): low-protein diet containing limiting amino acids (i.e., Lys, Met, Thr and Trp) at NRC (2012) levels; LPV: LP containing Val at NRC level; LPI: LP containing Ile at NRC level; LPVI: LP containing Val and Ile at NRC levels. The values are means ± standard error of the mean. *n* = 8. ^a,b,c^ Among groups, the means with different superscript letter(s) are different (*p* ≤ 0.05). ^#^ *p* ≤ 0.1 LPVI vs. PC, ^ɸ^ *p* ≤ 0.1 LPVI vs. LPV, ^$^ *p* ≤ 0.1 LPI vs. LP.

Overall, the effect of diet on gene expression of hepatic *IGF-1*, insulin-like growth factor binding protein 1 (*IGFBP1*), activating transcription factor 4 (*ATF4*), peroxisome proliferator-activated receptor gamma (*PPARγ*), and protein kinase C alpha (*PRKCA*) was significant (Figure 6A–E). Relative to PC, the gene expression of *IGF-1* was lower in LP (Figure 6A; *p* < 0.01), but the abundance of *IGF-1* transcript was not different for LPV and LPVI vs. PC. Compared with PC and LPV, the mRNA abundance of *IGF-1* was lower in LPI (Figure 6A). The mRNA abundance of *IGFBP1* was not different between PC and LP (Figure 6B). LPV and LPVI had lower mRNA abundance of *IGFBP1* than LP. LPI had higher *IGFBP1* gene expression than PC and that tended to be higher than LP (Figure 6B). No differences in the mRNA abundance of *ATF4* and *PPARγ* were detected when LP was compared to PC (Figure 6C,D). In comparison with LP, the mRNA abundance of *ATF4* and *PPARγ* was decreased in LPVI (Figure 6C,D). Further, relative to PC, the gene expression of *PPARγ* was reduced in LPVI (Figure 6D; *p* < 0.01). LPI had lower mRNA abundance of *PPARγ* compared with all other groups (Figure 6D). Although there were no differences between LP and PC on the mRNA abundance of *PRKCA*, that was higher in LPVI compared with PC (Figure 6E). LPI had lower mRNA abundance of *PRKCA* than LPVI (Figure 6E). While the effect of the diet on the mRNA abundance of casein kinase 2 alpha 1 (*CSNK2A1*) tended to be significant, no differences were detected among treatments (Figure 6F). No differences among treatments were detected for the mRNA abundance of hepatic general control non-derepressible-2 (*GCN2*) (Figure 6G) and mechanistic target of rapamycin (*mTOR*) (Figure 6H).

### 2.6. Plasma Metabolomics

**Figure 7:**
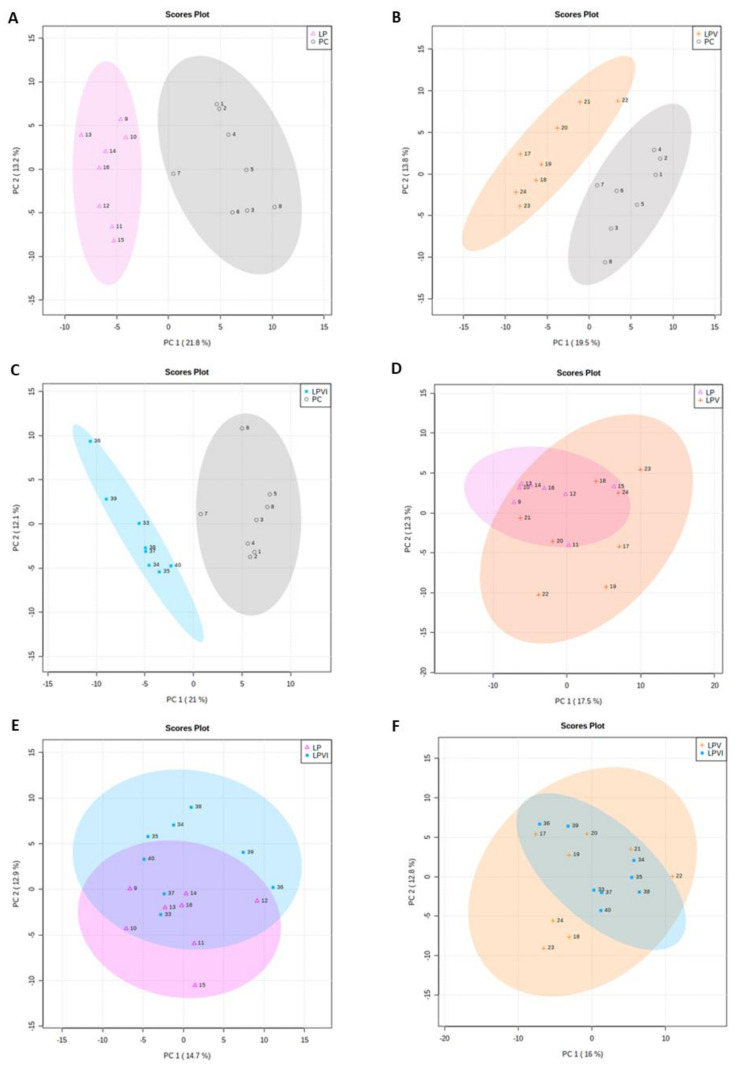
Principal component analysis (PCA) score plots of plasma metabolites in nursery pigs fed with very-low-protein diets supplemented with Ile, Val, or combination of both. (**A**) LP vs. PC, (**B**) LPV vs. PC, (**C**) LPVI vs. PC, (**D**) LPV vs. LP, (**E**) LPVI vs. LP, and (**F**) LPVI vs. LPV. PC (positive control): standard-protein diet; LP (negative control): low-protein diet containing limiting amino acids (i.e., Lys, Met, Thr and Trp) at NRC (2012) levels; LPV: LP containing Val at NRC level; LPI: LP containing Ile at NRC level; LPVI: LP containing Val and Ile at NRC levels. Each shape represents a pig. *n* = 8.

The principal component analysis (PCA) score plots for plasma metabolites are shown in Figure 7A–F. The plasma metabolites for pigs fed with PC diet were clearly separated from LP, LPV, LPVI, and LPI (Figure 7A–C and Supplementary Figure S1A). The separation of the scores is indicative of differences or variations in plasma metabolites identified among experimental groups (i.e., the clearer separation indicates the greater differences between groups or vice versa). The principal component 1 (PC1) indicated 21.8, 19.5, 21, and 21.5% variation and the PC2 showed 13.2, 13.8,12.1, and 12.8% variation between PC vs. LP, PC vs. LPV, PC vs. LPVI, and PC vs. LPI, respectively (Figure 7A–C and Supplementary Figure S1A). Although there was a cross-distribution for LPV vs. LP, LPVI vs. LP, and LPVI vs. LPV, little separations were detected among these comparisons (Figure 7D,E). No clear separations were observed in plasma metabolites for LPI vs. LP, LPI vs. LPV, and LPVI vs. LPI (Supplementary Figure S1B–D).

**Figure 8:**
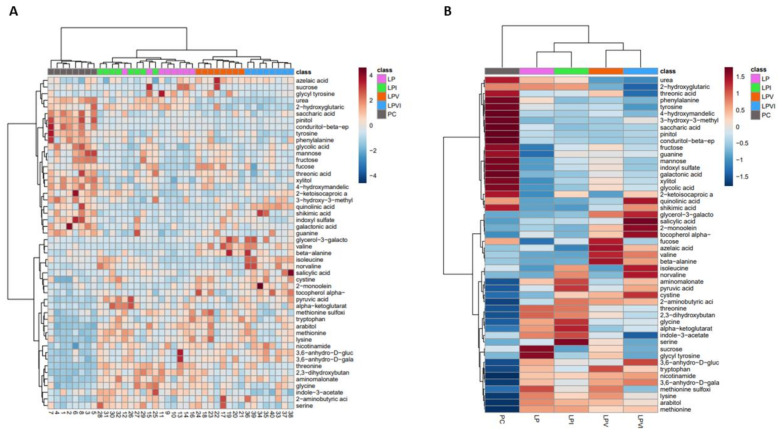
Heatmap analysis of plasma metabolites in nursery pigs fed with very-low-protein diets supplemented with Ile, Val, or combination of both. Hierarchical clustering of the top 50 significant plasma metabolites among: (**A**) individual pigs and (**B**) pigs fed with PC, LP, LPI, LPV, and LPVI. PC (positive control): standard-protein diet; LP (negative control): low-protein diet containing limiting amino acids (i.e., Lys, Met, Thr and Trp) at NRC (2012) levels; LPV: LP containing Val at NRC level; LPI: LP containing Ile at NRC level; LPVI: LP containing Val and Ile at NRC levels. The metabolites are shown in rows, and pigs or experimental groups are shown in columns (Figure 8A,B, respectively). The dark red or blue is corresponding to the magnitude of difference when compared with the average value. The dendrogram on the left side of the heatmap shows both the similarity and the order that the clusters were formed. *n* = 8.

The top 50 significant metabolites among groups were plotted for individual pigs (Figure 8A) and experimental groups (Figure 8B). The plasma metabolome of PC was associated with a relative increase in carbohydrate or carbohydrate derivative, AA, nucleotide, and metabolites related with protein and AA metabolism compared to the rest of the groups (Figure 8B). LPV increased the relative levels of azelaic acid involved in lipid metabolism and valine and beta-alanine (Figure 8B). LPVI showed a considerable increase in the level of metabolites that are either carbohydrate derivative, vitamin (α-tocopherol), AA (Ile, cystine and norvaline), or related with AA and lipid metabolism.

**Figure 9:**
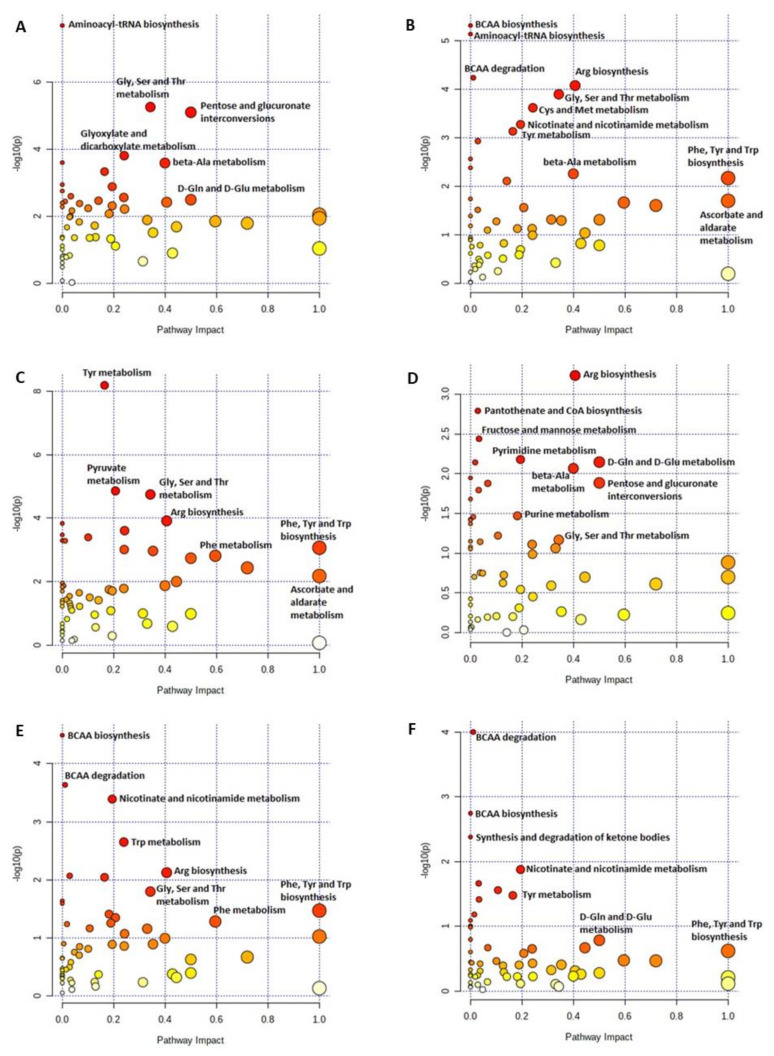
Pathway analysis map of plasma metabolites in nursery pigs fed with very-low-protein diets supplemented with Ile, Val, or combination of both. (**A**) LP vs. PC, (**B**) LPV vs. PC, (**C**) LPVI vs. PC, (**D**) LPV vs. LP, (**E**) LPVI vs. LP, and (**F**) LPVI vs. LPV. PC (positive control): standard-protein diet; LP (negative control): low-protein diet containing limiting amino acids (i.e., Lys, Met, Thr and Trp) at NRC (2012) levels; LPV: LP containing Val at NRC level; LPI: LP containing Ile at NRC level; LPVI: LP containing Val and Ile at NRC levels. Each circle is obtained from topology analysis representing a metabolic pathway with the scores. The *x*-axis indicates the pathway impact, and the *y*-axis shows the pathway enrichment. The circle size depends on its impact while its color is based on its *p* value (i.e., greater circle size shows higher pathway impact, while darker color circles demonstrate more significant changes of metabolites and pathway enrichment). *n* = 8.

The metabolic pathway enrichment analysis for LP vs. PC showed significant changes in the glycine–serine–threonine (Gly-Ser-Thr) metabolism, pentose and glucuronate interconversion, glyoxylate and dicarboxylate metabolism, D-glutamine (D-Gln) and D-glutamate (D-Glu) metabolism, and beta-alanine (β-Ala) metabolism (Figure 9A). Compared to PC, the BCAA metabolism, ascorbate and aldarate metabolism, arginine (Arg) biosynthesis, and other AA metabolisms (e.g., phenylalanine [Phe], tyrosine [Tyr] and Trp or Gly, Ser and Thr) were influenced by LPV and LPVI (Figure 9B,C). Similarly, LPV and LPVI mostly influenced the AA metabolism when compared to LP or to one another (Figure 9D–F). The pathway analyses for LPI vs. PC, LPI vs. LP, LPI vs. LPV, and LPVI vs. LPI are shown in Supplementary Figure S2A–D.

The significantly altered plasma metabolites among dietary groups are shown in Table 3 and the non-significant plasma metabolites are given in Supplementary Table S2. Regarding the metabolites for carbohydrates, their metabolism, and derivatives, LP reduced sorbitol, tagatose, fructose, mannose, glucose, and melibiose relative to PC. However, there were no differences for these metabolites when LPI, LPV, and LPVI were compared to either PC or LP (Table 3). Although no differences were detected between PC and LP for the abundance of cellobiose and pyruvic acid, LPVI and LPI had lower cellobiose, but higher pyruvic acid than PC. Regarding the metabolites for protein metabolism and AA, no differences were detected for LP vs. PC on levels of urea, creatinine, Phe, Tyr, and cystine. However, LPV and LPVI had lower levels of urea, creatinine, Phe, and Tyr and only LPVI had higher levels of cystine relative to PC. Relative to PC, LP had lower abundance of Val, Gln, Ile, Leu, shikimic acid, 2-ketoisocaproic acid, 3-hydroxy-3-methylglutaric acid, 3-hydroxypropionic acid, homocysteine, and indoxyl sulfate and had higher levels of 5-aminovaleric acid, α-aminoadipic acid, and α-ketoglutarate. However, LPV and LPVI had a higher level of Val than PC and their plasma levels of Gln, Leu, shikimic acid, homocysteine, indoxyl sulfate, 5-aminovaleric acid, α-aminoadipic acid, and α-ketoglutarate are the same as PC. The levels of 2-ketoisocaproic acid and 3-hydroxy-3-methylglutaric acid for LPVI and 3-hydroxypropionic acid for LPV were not different than PC.

**Figure 10:**
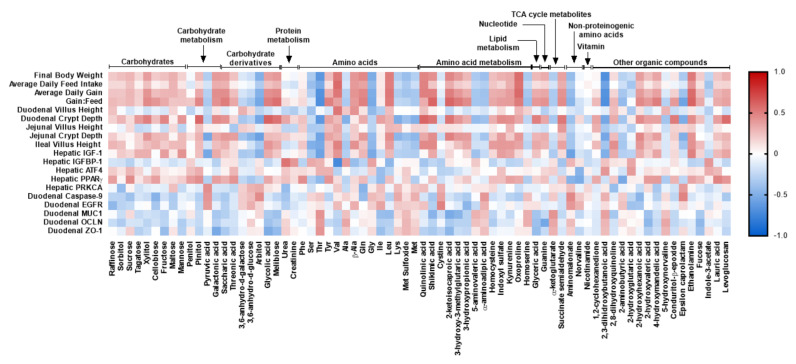
Pearson correlation analysis between significantly changed plasma metabolites, growth performance, gut morphology, and gene expression of markers associated with gut development and hepatic IGF-1 signaling pathway in nursery pigs fed with very-low-protein diets supplemented with Ile, Val, or combination of both. The color of the squares is based on the Pearson coefficient distribution: red represents a positive correlation (*p* ≤ 0.05), blue represents a negative correlation (*p* ≤ 0.05), and white is indicative of a non-significant correlation (*p* > 0.05). *IGFBP1*: insulin-like growth factor binding protein 1; *ATF4*: activating transcription factor 4; *PPARγ*: peroxisome proliferator-activated receptor gamma; *PRKCA*: protein kinase C alpha; *EGFR*: epidermal growth factor receptor; *MUC1*: mucin 1; *OCLN*: occludin; *ZO1*: zonula occludens 1. *n* = 8.

A correlation analysis showing the relationship between the significantly changed plasma metabolites among dietary groups and growth measurements, gut morphology, and gene expression of markers associated with gut development and hepatic IGF-1 signaling pathway is shown in Figure 10. Among the AA, Val, Tyr, β-Ala, Gln, and Leu levels were positively correlated with growth characteristics including final BW, ADG, ADFI, and G:F ratio (Figure 10; *p* < 0.05). While Thr abundance was negatively correlated with growth performance and gut morphology, the positive correlation was found between Thr and the gene expression of duodenal *MUC1*, *OCLN*, and *ZO1* (Figure 10; *p* < 0.05). Plasma Val, Gln, and Leu levels were positively correlated with gut morphology and the hepatic *IGF-1* gene expression and negatively correlated with hepatic *IGFBP1* mRNA abundance (Figure 10; *p* < 0.05). Further, metabolites of AA’s metabolism including quinolinic acid, shikimic acid, 2-ketoisocaproic acid, 3-hydroxy-3-methylglutaric acid, 3-hydroxypropionic acid, homocysteine, indoxyl sulfate, kynurenine, and oxoproline were positively correlated with growth performance measures, duodenal and jejunal crypt depth, ileal villus height, and hepatic *IGF-1*. This is while 5-aminovaleric acid and α-aminoadipic acid were negatively correlated with the above parameters. Carbohydrates and their derivatives including sorbitol, tagatose, xylitol, fructose, mannose, pinitol, galactonic acid, saccharic acid, threonic acid, glycolic acid, and melibiose were also positively correlated with the above parameters, but were negatively linked with duodenal *caspase 9*, *MUC1*, *OCLN*, and *ZO1*.

## 3. Discussion

Very-low-protein diets impair the growth of pigs and the supplementation of the first four LAA (i.e., Lys, Met, Thr, Trp) fails to recover the negative effects of these diets on growth performance [4,5,6,7]. Therefore, the deficiency of other AA is likely limiting the growth of pigs fed with VLP diets. Hence, this study aimed to determine the effects of Val, Ile, and combination of both on growth performance, intestinal morphology and development, hepatic *IGF-1* expression, and plasma metabolome in young pigs fed with VLP diets. In this study we showed that: (1) supplementation with a mixture of Val and Ile at NRC levels into the VLP diets (i.e., LPVI) recovered the negative effects of these diets on growth performance (e.g., ADG, BWG, G:P, etc.) and measurements (e.g., body length, etc.), which might be due to improved FI, gut development, and hepatic *IGF-1* expression, but not on feed efficiency (G:F ratio) which the latter might be explained by increased thermal radiation in this group; (2) LPVI restored the impaired gut morphology (e.g., duodenal villus width and crypt depth and ileal villus height and villus width) of pigs fed with VLP diets likely through enhanced mRNA abundance of gut *EGFR* and *caspase 9*; (3) both LPV and LPVI recovered the reduced transcript of hepatic *IGF-1* in pigs fed with VLP diets and reduced the mRNA abundance of *IGFBP1* in the liver and LPVI reduced the hepatic transcript of *ATF4*; and (4) LPV and LPVI recovered the reduced plasma Val, Gln, and Leu concentrations in pigs fed with VLP diet and reduced Thr concentration. The concentrations of Val, Gln, and Leu were positively correlated with gut morphology and the hepatic *IGF-1* gene expression and negatively correlated with hepatic *IGFBP1* mRNA abundance, but the level of plasma Thr was negatively correlated with gut morphology and hepatic *IGF-1* gene expression. Altogether, supplementation with a combination of Val and Ile into the VLP diets at levels recommended by NRC (2012), annulled the depressed growth performance of pigs fed with these diets likely through improved gut development, hepatic IGF-1 expression and bioavailability, and plasma metabolomics profile. 

In the current study, as expected, VLP diet reduced BW and ADG; however, supplementing VLP diets with a combination of Val and Ile at NRC levels (i.e., LPVI) recovered the growth parameters of young pigs fed with these diets. Similarly, we and others previously showed that feeding pigs with VLP diets containing the first four LAA negatively influence the growth performance of pigs [4,5,6,7], but adding Val alone or a combination of Val and Ile to these diets improves the growth [16,17,18,19,20,21]. Supplementing Ile alone to VLP diets did not improve the final BW and aggravated ADG in the present study. Likewise, others showed that Ile supplementation to VLP diets reduced weight gain and feed efficiency and depressed the growth rate in gilts [16] and growing pigs [17]. The improved performance following supplementation of a mixture of Val and Ile, but not with Ile alone can be attributed to changes in the FI. Here, we showed that LPVI completely recovered the FI, but LPI reduced the FI in animals fed with protein-restricted diets. Likewise, other studies showed that supplementation with Ile aggravates the reduced FI in VLP-fed pigs [16,17], but a mixture of Val and Ile improves the FI in pigs fed with SLP diets [22,36]. Although supplementing the mixture of Val and Ile improved the final BW and ADG in the current study, it did not change the feed efficiency, which might be due to a higher thermal radiation and energy loss in this group. Others reported a lower feed efficiency in pigs fed with SLP diets supplemented with Ile and Val on the ideal protein ratio basis [36]. Determination of the mechanisms by which Ile and Val individually or in combination regulate FI and energy expenditure may shed light on our understanding on the role of these AA in the regulation of growth. Little is understood about how dietary Val or a combination of Val and Ile could improve the growth of animals fed with protein-restricted diets. It has been shown that increasing the dose of dietary Val alone influences the circulating and tissues AA and metabolites profile in pigs fed with SLP diets [25]. Further, supplementation of Val into the standard-protein diet, has been reported to improve the AA utilization in growing pigs which may result in higher availability of AA for protein synthesis and growth [27]. Growth, health, and disease regulation are critically dependent upon the availability of AA [37]. Here, the metabolomics data showed an increase in plasma Val and β-Ala levels following the supplementation with Val or Val and Ile into the VLP diets. Val has been shown to play a critical role in growth, gut health, and protein accretion [38] while β-Ala improves endurance performance and lean body mass in human subjects [39]. Given the positive correlation between plasma Val and β-Ala with growth performance in the current study, the role of these metabolites seems to be critical under protein malnutrition. Whether modulation of growth by dietary Val and Ile (individually or combined) in pigs fed with VLP diets is related to changes in gut development, hepatic IGF-1 expression, and blood metabolites profile is poorly understood. 

Improved intestinal morphology and more likely the optimal function of small intestine can be characterized with measures such as higher villus height [12]. In the current study we showed that VLP diets reduced duodenal villus width and crypt depth, jejunal crypt depth, and ileal villus height and villus width. Similarly, a severe reduction in dietary protein negatively influenced some of morphometric indices in young [40] and adult pigs [41]. Here, for the first time, we showed that adding a mixture of Val and Ile to VLP diets restored the impaired gut morphology measures including duodenal villus width and crypt depth and ileal villus height and villus width. Further, we showed that LPI decreased the duodenal villus height, villus width, and crypt depth, jejunal crypt depth and ileal villus height, villus width and crypt depth. This might be linked with reduced plasma Thr, but recovered concentration of Val, Gln, and Leu in LPVI pigs. In this study, we showed a positive correlation between gut morphology and the levels of plasma Val, Gln, and Leu and a negative correlation between plasma Thr and gut morphology. Further research is required to explore the role of these AA in gut development. Improved intestinal morphology in LPVI pigs versus impaired gut development measures in LPI animals is likely contributing to improved and depressed growth in these pigs, respectively. Little is understood about how BCAA and in particular, Val and Ile, modulate the gut morphology. In that context, the association between dietary BCAA and epithelial cells proliferation and apoptosis has been overlooked. In this study, for the first time, we showed a higher expression of *EGFR* and *caspase 9* in the duodenum of pigs fed with VLP diets supplemented with both Val and Ile. EGFR is involved in the regulation of cell proliferation and epithelial cell growth [42], whereas caspase 9 is considered a marker for cell apoptosis. The higher expression of intestinal *EGFR* and *caspase 9* in LPVI pigs is suggestive of a greater epithelial cells’ proliferation and apoptosis or cell turnover in these animals. The restored gut morphology in LPVI pigs is likely due to a higher expression of *EGFR* and *caspase 9* in the gut. Further research is warranted to characterize the epithelial cells proliferation and apoptosis signaling pathways induced by Val and Ile.

Liver-derived IGF-1 is the major source for circulating IGF-1, which is involved in many physiological processes including the growth regulation [43]. Feeding pigs with VLP diets reduced the hepatic transcript of *IGF-1*. In line with this finding, we and others previously demonstrated that VLP diets reduce the concentration of blood IGF-1 in pigs [4,44,45]. This might be attributed to lower plasma Val, Ile, Gln, and Leu concentrations in protein-restricted pigs seen in this study and our previous work [4,5,8]. There is evidence that several AA such as Leu and Gln are involved in IGF-1 secretion [46,47]. Further studies on the direct effects of these AA on hepatic IGF-1 expression and its circulating concentration are warranted. Moreover, it has been shown that protein restriction increases the mRNA abundance of hepatic *IGFBP1* [48], which is a binding protein that inhibits the bioavailability of IGF-1 [49]. Adding Val or a combination of Val and Ile to VLP diets did recover the level of *IGF-1* transcript and reduced the mRNA abundance of *IGFBP1* whereas supplementing Ile reduced the mRNA abundance of *IGF-1* and increased the *IGFBP1* gene expression. This might be associated with recovered levels of plasma Val, Gln, and Leu concentrations in LPV and LPVI groups, but not LPI pigs. Here, we showed a positive correlation between concentrations of Val, Gln, and Leu and hepatic *IGF-1* gene expression, but a negative correlation with hepatic *IGFBP1* mRNA abundance. The higher hepatic *IGF-1* expression in VLP diets supplemented with Val and mixture of Val and Ile could be due to improved blood AA profile of these pigs as shown in their plasma metabolome. An increased transcript of hepatic *IGF-1* and its availability in LPVI and LPV pigs can play a role in improved growth of these animals while the reduced hepatic *IGF-1* transcript and its bioavailability in LPI can contribute to their reduced growth. The underlying intracellular mechanisms by which Val or mixture of Val and Ile induce hepatic *IGF-1* are not fully understood and require further investigation. Protein dilution and AA insufficiency sensed by various organs including the liver through unchanged tRNA, GCN2, eukaryotic translation initiation factorα and ATF4 and alter energy balance and growth [50]. Here, we showed that LPVI reduced the hepatic transcript of *ATF4* and *PPARγ* but increased that of *PRKCA*. ATF4 is involved in fibroblast growth factor 21 (FGF21) gene regulation in multiple organs including the liver [51]. The lower transcript of hepatic ATF4 in pigs fed with LPVI is suggestive of a lower production of FGF21 in the liver of these pigs. It has been shown that transgenic mice overexpressing FGF21 have lower concentrations of blood IGF-1 and are significantly smaller than wild-type mice [52]. Therefore, we speculate that a lower expression of hepatic FGF21 is contributing to higher circulating IGF-1 concentration in LPVI pigs and their improved growth. However, further investigations are needed to better understand the links between BCAA sensing in the liver and its influence on IGF-1 concentration and availability.

## 4. Materials and Methods

### 4.1. Animals and Housing

The experimental procedures were approved by Oklahoma State University’s Institutional Animal Care and Use Committee (protocol# IACUC-20-54; approved 28 August 2020). A total of 40, three-week-old (6.29 ± 0.14 kg), weaned barrows (Duroc sire line and Large White X Landrace dam; Seaboard, Hennessey, OK, USA) were housed in an environmentally controlled facility. The room temperature was set at 30 °C in week 1 of study and was decreased weekly by 1 °C. A 12 h light/half-light cycle was applied as lighting program. Feed and water were provided ad libitum using single-hole stainless steel feeders and cup waterers (Aqua ChiefTM, Hog Slat Inc., Newton Grove, NC, USA) with a single 1/2″ nipple (Lixit^®^ Nipple Waterer—L-70, QC Supply, Schuyler, NE, USA). All pigs were fed, and their water buckets were filled once a day at 0800.

### 4.2. Diets and Experimental Design

Following an initial one week of adaptation, all pigs were weighed, and weight matched. For the duration of five weeks, pigs were then individually housed and randomly allocated to five dietary treatments [average BW: 6.75 ± 0.14 kg; 8 pigs/group] including: (1) PC: [53]; (2) LP; (3) LPV; (4) LPI; and (5) LPVI. National Swine Nutrition Guide (Version 2.1 Metric, ©2012 U.S. Pork Center of Excellence, Des Moines, IA, USA) was used to formulate the diets. Phase feeding approach was applied to accurately meet the nutrient requirements of the pigs. The nursery phase 1 (N1) diet was provided during the adaptation period for one week (days 1–7). Then, pigs were fed with nursery phase 2 diet (N2) from days 8 to 21 and the nursery phase 3 diets (N3) from days 22 to 42 according to NRC recommendations. The ingredients and calculated composition of diets are given in Table 4. By manipulating the amount of corn, soybean meal, and corn starch, all diets were kept isocaloric. Moreover, using L-Ala, LP, LPV, LPI, and LPVI were kept isonitrogenous. All the other ingredients were kept consistent among dietary treatments (Table 4).

### 4.3. Feed and Water Intake, Body Weight, and Growth Measurements

FI and WI were measured daily. Further, FI at 3, 6, 9, 12, and 24 h after offering the daily meal was measured biweekly. Body weight and physical growth measurements including body length, wither height, and heart girth of all pigs were measured weekly. ADG (final BW—initial BW/experimental days), ADFI (cumulative FI/experimental days), ADPI (cumulative PI [FI × %CP]/experimental days), ADWI (cumulative WI/experimental days), G:F (ADG/ADFI), G:P (ADG/ADPI), and W:F (ADWI/ADFI) were calculated accordingly. Moreover, weekly BWG, MFI, CFI, CPI, G:F, and G:P were calculated. 

### 4.4. Thermal Imaging

To measure the radiated heat from the body surface, thermal images were captured from each pig in seven-day intervals using a FLIR C2 compact thermal camera with emissivity set at 0.86 (focal length: 1.54 mm; thermal accuracy: ±2 °C; FLIR Systems, Boston, MA, USA). The room temperature was recorded daily to calculate the ambient temperature. All images were taken at ~1 m above each pig with one pig in each image and the focal point in the center of the back.

### 4.5. Feed, Blood, and Tissue Samples Collection

Following mixing the diets, feed samples were collected from several feed bags of each treatment (~1 kg), pooled, and stored at −20 °C until analysis. At the end of the experiment (day 35), pigs were fasted for eight hours (0000–0800). Following fasting blood samples collection, all pigs were fed for 1 h from their respective diets and blood samples were collected at 60 min (min) and 120 min after meal. Blood samples were collected via the jugular vein in the supine position into 3.0 mL lithium heparin containing tubes and 10.0 mL serum tubes (BD Vacutainer^®^, Franklin Lakes, NJ, USA). The collected blood samples were transferred to the laboratory on ice and centrifuged at 3000× *g* for 15 min at 4 °C. The plasma and serum samples were stored at −80 °C until further analysis. At 120 min after the meal test, all pigs were euthanized by CO_2_ asphyxiation method. Immediately after euthanasia the duodenum and liver samples were collected, snap-frozen in liquid N and stored at −80 °C until analysis. Moreover, gut samples from duodenum, jejunum, and ileum segments were collected and fixed in 10% formaldehyde for gut morphology assessment.

### 4.6. Diets Chemical Composition and Amino Acid Content Analysis

The experimental diets were analyzed for dry matter, CP, crude fat, crude fiber, calcium, phosphorus, and N by Servi-Tech laboratory (Dodge City, KS, USA) as we previously described [4,8,54]. Dietary AA concentration was quantified at Agricultural Experiment Station Chemical Laboratories (University of Missouri-Columbia, MO, USA) following previously published procedures [55]. The result of the above analysis is presented in Table 5.

### 4.7. Thermal Radiation Analysis

As we previously described [55], thermal images were analyzed using FLIR Research Studio software (FLIR Systems, Boston, MA, USA). For obtaining the dorsal surface mean body temperature, a rectangular shape was drawn in the region of interest (i.e., from shoulder to the rump; Supplementary Figure S3). Heat loss via thermal radiation (W/m^2^) was calculated as follows: σε (*T*_*s*_^4^−*T*_α_^4^). In this equation, *σ* is Stefan–Boltzmann constant (5.67 × 10^–8^ W/m^2^K^4^), ε is thermodynamic emissivity (0.86), T_s_ is the mean body surface temperature (kelvin), and T_α_ is the ambient temperature (kelvin).

### 4.8. H&E Staining and Gut Morphology Measurements

Gut segments were coated in paraffin and cut into 5 μm sections. Following staining, the sections with hematoxylin and eosin, using a BZ-X800E Keyence all-in-one fluorescence microscope (BZ-X710, IL, USA) at least, ten well-oriented villi and crypt in each section were considered to measure villus height and width, crypt depth and width, and muscle thickness [55]. Images were captured by Keyence BZ-X Viewer (Keyence Co. USA, Itasca, IL, USA) and analyzed using ImageJ software (https://imagej.nih.gov/ij/download.html; accessed on 13 June 2021).

### 4.9. Plasma Metabolomics

Plasma metabolomics was performed at West Coast Metabolomics Center (UC Davis, Davis, CA, USA) as we previously described [5,8]. Briefly, plasma samples were prepared and analyzed by gas chromatography (GC)—mass spectrometry (MS) using a time-of-flight mass spectrometer (GC-TOF MS; Leco Pegasus IV, St. Joseph, MI, USA). For data acquisition, an Agilent 690 GC with an automatic linear exchange (ALEX; Gerstel corporation, Linthicum, MD, USA) and cold injection system (CIS; Gerstel corporation, Linthicum, MD, USA) was used. The qualified metabolites were then reported as peak height. Using MetaboAnalyst 5.0 (http://www.metaboanalyst.ca/faces/ModuleView.xhtml; accessed on 4 September 2021) PCA, pathway impact analysis and hierarchical clustering analysis (heatmap) on the metabolites were performed.

### 4.10. RNA Isolation, Reverse Transcription, and Quantitative PCR (qPCR)

RNA isolation, reverse transcription, and quantitative PCR were performed as we previously described [4,56,57]. Following total RNA extraction, the RNA was quantified using a NanoDrop ND-1000 spectrophotometer (Thermo Fisher, Waltham, MA, USA). The thermocycler (T100TM Thermal Cycler, Bio-Rad, Hercules, CA, USA) was used to synthesize the complimentary DNA and a CFX96 real-time PCR detection system (Bio-Rad, Hercules, CA, USA) was used for real-time quantitative PCR (qPCR). To obtain the mRNA abundance of target and housekeeping (β-actin) [58] genes, specific primers (Supplementary Table S3) for following genes were obtained from previous studies or designed in-house: *MUC1* [59], mucin 2 (*MUC2*) [59], *EGFR* [60], insulin-like growth factor-1 receptor (*IGF-1R*) [61], *caspase-9* [62], *Ki-67* [63], *PPARγ* [64], *IGFBP1* [65], *IGF-1* [66], *mTOR* [67], *ATF4* [68], *CSNK2A1* [69], *OCLN* [70], *ZO1* [70], *PRKCA*, and *GCN2*. The qPCR program was followed by a melt curve program. Finally, the relative mRNA abundance of target genes was calculated using the 2^−∆∆CT^ method.

### 4.11. Statistical Analysis

Overall, growth performance data, AUC of thermal radiation, gut morphology, and qPCR data were analyzed with GLM procedure followed by Tukey post hoc to detect differences among treatment means (IBM SPSS Statistics Version 23, Armonk, NY, USA). For the hourly, daily, and weekly collected data including FI, WI, BW, BWG, MFI, CFI, CPI, G:F, G:P, and thermal radiation the mixed analysis was performed considering the diet, time, and the interaction of diet by time as fixed effects and the animal as a random variable in the model. Based on the smallest quantities of fit statistics for corrected Akaike Information Criterion and Bayesian Information Criterion, the modeling of covariance structure for the repeated measurements for each variable was performed. Differences among treatments were considered significant at *p* ≤ 0.05 and a trend at 0.05 < *p* ≤ 0.10.

For producing PCA plots and performing pathway and heatmap analyses on metabolomics data following interquartile range of data filtering and normalization by median, the analysis of variance (ANOVA) was performed using MetaboAnalyst 5.0 (https://www.metaboanalyst.ca/MetaboAnalyst/home.xhtml; accessed on 4 September 2021). Moreover, to obtain the mean values of metabolites for each treatment, all metabolites were tested for normal distribution using the Shapiro–Wilk test of normality, and the non-normal distributions were normalized using inverse distribution function (IDF-normal) in SPSS (IBM SPSS Statistics Version 23, Armonk, NY, USA). Then, all metabolites were analyzed using GLM procedure followed by Tukey post hoc to determine the differences among treatment means (IBM SPSS Statistics Version 23, Armonk, NY, USA). Differences were considered significant at *p* ≤ 0.05 and a trend at 0.05 < *p* ≤ 0.10. Furthermore, to assess the relationship of growth performance and qPCR data with significantly altered plasma metabolites, the Pearson correlation was performed using GraphPad software (Prism 7.05, San Diego, CA, USA).

## 5. Conclusions

To our knowledge, this is the first study assessing the links between Val/Ile-induced growth and gut development, hepatic IGF-1 expression, and plasma metabolomics profile in pigs fed with very-low-protein diets. Here, we showed that supplementation with a combination of Val and Ile into the protein-restricted diets at levels recommended by NRC (2012), annulled the depressed growth performance of pigs fed with these diets likely through improved FI, gut development, hepatic IGF-1 expression, and plasma metabolomics profile. A mixture of Val and Ile restored the impaired gut morphology of pigs fed with VLP diets likely through enhanced transcript of gut EGFR. Supplementation with Val alone or in combination with Ile not only recovered the reduced transcript of hepatic IGF-1 and plasma Val, Gln, and Leu concentrations, but also reduced the mRNA abundance of IGFBP1 in the liver of pigs fed with VLP diets. Further research is required to better understand the role of Ile and Val on epithelial cells turnover and the molecular pathways involved in secretion and bioavailability of IGF-1 under protein malnutrition.

## Figures and Tables

**Table 1:** Growth measurements of nursery pigs fed with very-low-protein diets supplemented with Ile, Val, or combination of both.

Measurements	Diets ^1^						
	PC	LP	LPV	LPI	LPVI	SEM ^2^	*p*-Value
Initial BW ^1^, kg	6.76	6.76	6.74	6.73	6.76	0.14	1
Final BW ^1^, kg	23.47 ^a^	16.24 ^bc^	19.35 ^ab^*	13.66 ^c^	21.74 ^a^	0.75	<0.01
ADG ^1^, kg/d	0.48 ^a^	0.27 ^c^	0.36 ^bc^	0.20 ^d^	0.42 ^ab^	0.02	<0.01
ADFI ^1^, kg/d	0.75 ^ab^	0.61 ^bc^	0.69 ^ab^	0.46 ^c$^	0.79 ^a^	0.02	<0.01
ADPI ^1^, kg/d	0.14 ^a^	0.08 ^bc^	0.09 ^b^	0.06 ^c^	0.10 ^b&^	0.005	<0.01
ADWI ^1^, L/d	2.01 ^a^	1.58 ^ab^	1.64 ^a^	1.18 ^b^	1.87 ^a^	0.07	<0.01
G:F ^1^, kg/kg	0.64 ^a^	0.44 ^cd^	0.53 ^b^	0.42 ^d^	0.51 ^bc&^	0.01	<0.01
G:P ^1^, kg/kg	3.40 ^ab^	3.36 ^b^	3.93 ^a^*	3.29 ^b^	3.96 ^a#^	0.08	<0.01
W:F ^1^, L/kg	2.48	2.74	2.38	2.6	2.41	0.09	0.7
Final body length, m	0.71 ^a^	0.65 ^b^	0.65 ^b^	0.62 ^b^	0.68 ^ab@^	0.01	<0.01
Final heart girth, m	0.61 ^a^	0.55 ^bc^	0.58 ^ab^	0.52 ^c^	0.60 ^ab^	0.01	<0.01
Final wither height, m	0.42 ^ab^	0.39 ^bc§^	0.40 ^ab^	0.37 ^c^	0.42 ^a^	0.004	<0.01

^1^ PC (positive control), standard-protein diet; LP (negative control), low-protein diet containing limiting amino acids (i.e., Lys, Met, Thr and Trp) at NRC (2012) levels; LPV: LP containing Val at NRC level; LPI: LP containing Ile at NRC level; LPVI: LP containing Val and Ile at NRC levels; BW: body weight; ADG: average daily gain; ADFI: average daily feed intake; ADPI: average daily protein intake; ADWI: average daily water intake; G:F: gain:feed ratio; G:P: gain:protein ratio; W:F: water:feed ratio. The values are means, *n* = 8. ^2^ SEM: standard error of the mean. ^a,b,c,d^ Within each row, the values with different superscript letter(s) are different (*p* ≤ 0.05). ^§^ *p* ≤ 0.1 LP vs. PC, * *p* ≤ 0.1 LPV vs. PC, ^#^ *p* ≤ 0.1 LPVI vs. PC, ^$^ *p* ≤ 0.1 LPI vs. LP, ^&^ *p* ≤ 0.1 LPVI vs. LP, ^@^ *p* ≤ 0.1 LPVI vs. LPI.

**Table 2:** Intestinal morphology of nursery pigs fed with very-low-protein diets supplemented with Ile, Val, or combination of both.

Measurements			Diets ^1^				
	PC	LP	LPV	LPI	LPVI	SEM ^2^	*p*-Value
Duodenum (μm)							
Villus height	525.0 ^ab^	533.9 ^ab^	589.6 ^a^	463.2 ^b^	549.4 ^ab^	12.3	0.01
Villus width	175.1 ^a^	144.5 ^bc^	151.4 ^abc^	136.2 ^c^	164.8 ^ab^	3.7	<0.01
Crypt depth	581.8 ^a^	366.3 ^c^	366.1 ^c^	379.3 ^c^	488.4 ^b^	16.6	<0.01
Crypt width	51.8	48.7	48.2	47.0	50.2	0.6	0.15
Muscular thickness	350.4 ^ab^	285.3 ^b^	355.8 ^abΨ^	367.0 ^a^	309.4 ^ab^	9.3	0.02
Villus height: Crypt depth	0.9 ^c^	1.4 ^ab^	1.6 ^a^	1.2 ^abc^	1.1 ^bc^	0.06	<0.01
Jejunum (μm)							
Villus height	390.5 ^ab^	390.1 ^ab^	391.9 ^ab^	340.5 ^b^	427.5 ^a^	8.6	0.02
Villus width	143.4	138.9	137.4	133.1	128.3	2.2	0.23
Crypt depth	321.7 ^a^	236.3 ^b^	255.6 ^b^	241.7 ^b^	262.4 ^ab#^	7.9	<0.01
Crypt width	48.9 ^ab^	46.6 ^ab^	52.9 ^a^	48.7 ^ab^	45.8 ^b^	0.8	0.05
Muscular thickness	286.6	287.5	314.4	314.0	303.0	6.4	0.47
Villus height: Crypt depth	1.2	1.6	1.6	1.4	1.6	0.06	0.12
Ileum (μm)							
Villus height	388.8 ^a^	319.4 ^bc^	362.5 ^ab^	286.0 ^c^	379.8 ^a^	9.0	<0.01
Villus width	143.6 ^a^	122.1 ^b^	136.1 ^ab^	121.7 ^b‡^	132.8 ^ab^	2.1	<0.01
Crypt depth	247.4	207.6	238.6	212.8	218.3	5.8	0.14
Crypt width	53.6 ^a^	50.3 ^ab^	51.9 ^ab^	47.7 ^b^	51.4 ^ab^	0.6	0.02
Muscular thickness	487.5	554.5	503.1	562.3	493.2	16.9	0.48
Villus height: Crypt depth	1.5 ^ab^	1.5 ^ab^	1.5 ^ab^	1.3 ^b^	1.8 ^a^	0.06	0.09

^1^ PC (positive control): standard-protein diet; LP (negative control): low-protein diet containing limiting amino acids (i.e., Lys, Met, Thr and Trp) at NRC (2012) levels; LPV: LP containing Val at NRC level; LPI: LP containing Ile at NRC level; LPVI: LP containing Val and Ile at NRC levels. The values are means, *n* = 8. ^2^ SEM: standard error of the mean. ^a,b,c^ Within each row, the values with different superscript letter(s) are different (*p* ≤ 0.05). ^#^ *p* ≤ 0.1 LPVI vs. PC, ^Ψ^ *p* ≤ 0.1 LPV vs. LP, ^‡^ *p* ≤ 0.1 LPI vs. LPV.

**Table 3:** Significantly different plasma metabolites in nursery pigs fed with very-low-protein diets supplemented with Ile, Val, or combination of both.

Metabolites	Diets ^1^					SEM ^2^	*p*-Value
	PC	LP	LPV	LPI	LPVI		
Carbohydrates							
Raffinose	1618	1508	1421	987 ^¥^	1078	82	0.05
Sorbitol	21,286 ^a^	18,143 ^b^	20,025 ^ab^	20,134 ^ab^	19,169 ^ab^	329	0.02
Sucrose	8391 ^ab^	8763 ^a^	7630 ^abc^	6370 ^c^	6853 ^bc^	236	<0.01
Tagatose	16,704 ^a^	14,922 ^b^	15,873 ^ab^	15,722 ^ab^	15,669 ^ab^	182	0.04
Xylitol	16,849 ^a^	15,570 ^b^	15,988 ^b^	15,834 ^b^	15,988 ^b^	107	<0.01
Cellobiose	2121 ^a^	1987 ^ab^	1876 ^b^	1872 ^b^	1889 ^ab#^	29	0.02
Fructose	1,001,056 ^a^	877,412 ^b^	942,126 ^ab^	925,428 ^ab^	933,236 ^ab^	10,741	<0.01
Maltose	3878 ^ab^	4073 ^a^	3824 ^ab^	3634 ^b^	3946 ^ab^	45	0.02
Mannose	169,383 ^a^	146,492 ^b^	158,060 ^ab^	154,729 ^ab^	153,144 ^ab#^	2090	<0.01
β-gentiobiose	2577 ^a^	2369 ^ab^	2325 ^ab^	2280 ^b^	2365 ^ab^	36	0.07
Glucose	2,828,669 ^a^	2,031,578 ^b^	2,250,149 ^ab^	2,379,230 ^ab^	2,278,446 ^ab^	92,284	0.08
Carbohydrate Metabolism							
Pentitol	947 ^a^	918 ^ab^	893 ^ab^	883 ^ab^	868 ^b^	9	0.04
Pinitol	10,367 ^a^	8931 ^b^	8064 ^b^	8339 ^b^	8666 ^b^	169	<0.01
Pyruvic acid	11,135 ^b^	12,073 ^ab^	11,985 ^ab^	12,675 ^a^	12,744 ^a^	153	<0.01
Galactonic acid	1755 ^a^	1424 ^b^	1540 ^b^	1456 ^b^	1539 ^b^	28	<0.01
3-phosphoglycerate	700	479	732	568	654	33	0.09
Carbohydrate Derivatives							
Saccharic acid	5511 ^a^	5029 ^b^	4835 ^b^	4864 ^b^	4982 ^b^	58	<0.01
Threonic acid	60,141 ^a^	56,667 ^ab^	57,363 ^ab^	56,396 ^ab^	54,750 ^b^	510	0.01
3,6-anhydro-d-galactose	4281 ^b^	4576 ^ab§^	4656 ^a^	4565 ^ab¥^	4748 ^a^	41	<0.01
3,6-anhydro-d-glucose	3671 ^b^	4070 ^a^	4035 ^a^	4055 ^a^	4317 ^aɸ^	46	<0.01
Arabitol	38,140 ^b^	71,093 ^a^	61,908 ^a^	65,003 ^a^	62,405 ^a^	2591	<0.01
Glycolic acid	20,388 ^a^	18,494 ^b^	19,216 ^ab^*	18,755 ^b^	19,228 ^ab#^	162	<0.01
Melibiose	534 ^a^	491 ^b^	502 ^ab^	494 ^ab¥^	513 ^ab^	5	0.04
Gluconic acid	2857	2753	2765	2726	2703 ^#^	19	0.08
Glycerol-3-galactoside	6293	6158	6931	6401	7238	146	0.09
Xylonic acid	692	688	673	649	693	6	0.09
Protein Metabolism							
Urea	1,163,857 ^a^	1,086,906 ^ab^	944,967 ^c^	1,054,742 ^b^	949,939 ^c^	16,495	<0.01
Creatinine	347,148 ^a^	338,469 ^ab^	308,409 ^b^	336,729 ^ab^	310,338 ^b^	4446	<0.01
Amino Acids							
Phenylalanine	143,857 ^a^	139,217 ^ab^	135,209 ^b^	134,917 ^b^	135,149 ^b^	986	0.01
Serine	658,677 ^b^	668,391 ^ab^	647,477 ^b^	859,878 ^a$^	610,768 ^b^	25,129	<0.01
Threonine	285,059 ^c^	1,745,366 ^a^	1,050,660 ^b^	1,680,534 ^a^	1,046,112 ^b^	102,286	<0.01
Tyrosine	480,777 ^a^	452,008 ^ab§^	437,185 ^b^	441,220 ^b^	430,489 ^b^	4108	<0.01
Valine	933,798 ^b^	809,078 ^c^	1,051,075 ^a^	817,114 ^c^	1,042,142 ^a^	19,333	<0.01
Alanine	2,770,268 ^b^	4,403,453 ^a^	4,326,920 ^a^	4,529,566 ^a^	3,538,647 ^ab^	193,809	0.01
β-alanine	48,631 ^ab^	46,398 ^b^	51,255 ^a^	46,438 ^b^	50,943 ^a^	537	<0.01
Glutamine	518,564 ^a^	475,176 ^b^	499,561 ^ab^	484,307 ^ab¥^	488,913 ^ab^	4471	0.01
Glycine	1,469,737 ^b^	1,938,561 ^ab§^	1,600,625 ^ab^	2,071,491 ^a‡^	1,740,154 ^ab^	64,280	0.01
Isoleucine	573,556 ^b^	513,193 ^c^	507,862 ^c^	602,168 ^ab^	640,497 ^a@^	9216	<0.01
Leucine	765,367 ^a^	724,908 ^b^	741,675 ^ab^	717,864 ^b^	743,350 ^ab^	4401	<0.01
Lysine	110,936 ^b^	298,970 ^a^	274,360 ^a^	245,614 ^a^	237,811 ^ab#^	17,260	<0.01
Methionine sulfoxide	41,269 ^b^	68,702 ^a^	61,513 ^ab^	52,958 ^ab^	45,483 ^ab&^	3100	0.02
Methionine	55,080 ^b^	141,048 ^a^	119,972 ^a^	139,739 ^a^	128,910 ^a^	7690	<0.01
Glutamic acid	224,588	213,413	223,754	212,972	215,753	1737	0.07
Cystine	20,271 ^b^	23,298 ^b^	29,433 ^ab^	29,283 ^ab^	37,139 ^a^	1596	<0.01
Ornithine	340,342	321,653 ^§^	324,761	321,314 ^¥^	328,481	2370	0.08
Amino Acids Metabolism							
Quinolinic acid	2644 ^ab^	2368 ^b^	2314 ^b^	2334 ^b^	3011 ^a^	65	<0.01
Shikimic acid	4903 ^a^	4222 ^b^	4404 ^ab^*	4136 ^b^	4513 ^ab^	69	<0.01
2-ketoisocaproic acid	21,931 ^a^	18,660 ^c^	19,026 ^bc^	20,444 ^ab¥^	20,842 ^a^	255	<0.01
3-hydroxy-3-methylglutaric acid	739 ^a^	690 ^b^	688 ^b^	687 ^b^	706 ^ab^	6	0.01
3-hydroxypropionic acid	25,691 ^a^	23,118 ^b^	23,401 ^ab^*	22,481 ^b^	23,119 ^b^	304	<0.01
5-aminovaleric acid	12,986 ^b^	26,827 ^a^	20,598 ^ab^	22,082 ^ab^	19,011 ^ab^	1390	0.02
α-aminoadipic acid	31,254 ^b^	53,213 ^a^	49,317 ^ab^	52,183 ^ab¥^	46,665 ^ab^	2599	0.04
Homocysteine	3143 ^a^	2770 ^b^	2900 ^ab^	2809 ^b^	2964 ^ab^	35	<0.01
Indoxyl sulfate	2349 ^a^	1919 ^b^	2137 ^ab^	2051 ^ab¥^	2026 ^ab#^	40	0.01
Kynurenine	2662 ^a^	2436 ^ab^	2422 ^ab^	2293 ^b^	2517 ^ab^	34	<0.01
Oxoproline	1,535,721 ^a^	1,244,323 ^ab^	1,253,116 ^ab^	1,089,477 ^b^	1,258,715 ^ab^	46,704	0.04
3-phenyllactic acid	1829	1727	1656 *	1692	1692	22	0.09
Glycyl proline	1669	1616	1567	1204 ^¥^	1374	59	0.07
Homoserine	1001 ^b^	1367 ^ab^	1314 ^ab^	1452 ^a^	1229 ^ab^	52	0.05
Lipid Metabolism							
Glyceric acid	76,338 ^a^	71,394 ^b^	73,748 ^ab^	73,489 ^ab^	73,635 ^ab^	432	<0.01
2-monoolein	1966	1962	2023	1912	2090 ^@^	21	0.08
Azelaic acid	1357	1359	1396	1312	1406 ^@^	12	0.08
Nucleotide							
Uracil	9387	8502 ^§^	8673	8608	8745	108	0.06
Guanine	5101 ^a^	4701 ^b^	4824 ^ab^	4849 ^ab^	4800 ^ab^	41	0.02
Cytidine	784	715 ^§^	765	733	747	8	0.07
TCA Cycle Metabolites							
α-ketoglutarate	2642 ^b^	3026 ^a^	2781 ^ab^	3005 ^a^	2798 ^ab^	44	0.02
Succinate semialdehyde	2801 ^a^	2637 ^ab^	2601 ^ab^	2517 ^b^	2711 ^ab^	30	0.02
Non-Proteinogenic Amino Acids							
Aminomalonate	37,321 ^c^	66,126 ^ab^	51,318 ^bc^	77,691 ^a^	69,751 ^ab^	3249	<0.01
Norvaline	5562 ^b^	5408 ^b^	5388 ^b^	6350 ^a^	6496 ^a^	96	<0.01
Vitamins							
α-tocopherol	2187	2259	2244	2196	2357 ^#@^	21	0.06
Nicotinamide	2561 ^b^	4729 ^ab^	4643 ^ab^	4459 ^ab^	5412 ^a^	312	0.04
Other Organic Compounds							
1,2-cyclohexanedione	4280 ^a^	4053 ^ab^	3855 ^b^	3866 ^ab¥^	3945 ^ab^	50	0.05
2,3-dihydroxybutanoic acid	1319 ^c^	5249 ^a^	3631 ^ab^	4801 ^ab^	3177 ^bc^	311	<0.01
2,8-dihydroxyquinoline	672 ^a^	574 ^b^	621 ^ab^	597 ^ab¥^	600 ^ab#^	10	0.01
2-aminobutyric acid	33,956 ^b^	61,221 ^ab^	77,741 ^a^	89,807 ^a^	80,154 ^a^	4739	<0.01
2-hydroxyglutaric acid	7064 ^a^	7010 ^ab^	6605 ^bcΨ^	6960 ^ab^	6543 ^c^	57	<0.01
2-hydroxyhexanoic acid	3205 ^a^	2891 ^ab^	2807 ^b^	2806 ^b^	2872 ^ab#^	46	0.02
2-hydroxyvaleric acid	5754 ^a^	5388 ^b^	5562 ^ab^	5440 ^ab¥^	5499 ^ab^	40	0.04
4-hydroxymandelic acid	1718 ^a^	1584 ^b^	1573 ^b^	1555 ^b^	1564 ^b^	15	<0.01
5-hydroxynorvaline	10,602 ^b^	15,180 ^ab§^	15,461 ^a^	14,879 ^ab¥^	15,096 ^ab#^	564	0.02
Conduritol-β-epoxide	23,513 ^a^	20,386 ^b^	18,477 ^b^	19,762 ^b^	19,819 ^b^	361	<0.01
Epsilon caprolactam	5778 ^b^	6783 ^a^	6686 ^a^	6744 ^a^	6818 ^a^	96	<0.01
Ethanolamine	10,934 ^a^	5610 ^b^	7176 ^ab^	5995 ^b^	6924 ^ab^	559	0.01
Fucose	8445 ^a^	5859 ^b^	7887 ^abΨ^	6682 ^ab^	6859 ^ab^	273	0.02
Indole-3-acetate	4093 ^ab^	4451 ^a^	3554 ^ab^	4380 ^a^	2880 ^b#^	170	0.01
Lauric acid	12,353	11,819	12,050	11,571 ^¥^	11,568 ^#^	96	0.04
Levoglucosan	1443 ^a^	976 ^b^	949 ^b^	883 ^b^	1046 ^ab#^	53	<0.01
3-hydroxybutyric acid	16,574	15,869	16,664	15,665	15,323	184	0.08
Malic acid	22,806 ^ab^	23,563 ^a^	22,363 ^ab^	23,027 ^ab^	21,652 ^b^	225	0.07

^1^ PC (positive control): standard-protein diet; LP (negative control): low-protein diet containing limiting amino acids (i.e., Lys, Met, Thr and Trp) at NRC (2012) levels; LPV: LP containing Val at NRC level; LPI: LP containing Ile at NRC level; LPVI: LP containing Val and Ile at NRC levels. The values are mean peak height, *n* = 8. ^2^ SEM: standard error of the mean. ^a,b,c^ Within each row, the values with different superscript letter(s) are different (*p* ≤ 0.05). ^§^ *p* ≤ 0.1 LP vs. PC, * *p* ≤ 0.1 LPV vs. PC, ^¥^ *p* ≤ 0.1 LPI vs. PC, ^#^ *p* ≤ 0.1 LPVI vs. PC, ^$^ *p* ≤ 0.1 LPI vs. LP, ^@^ *p* ≤ 0.1 LPVI vs. LPI, ^Ψ^ *p* ≤ 0.1 LPV vs. LP, ^ɸ^ *p* ≤ 0.1 LPVI vs. LPV, ^‡^ *p* ≤ 0.1 LPI vs. LPV, ^&^ *p* ≤ 0.1 LPVI vs. LP.

**Table 4:** Ingredients and calculated chemical composition of experimental diets (as-fed basis).

	Diets ^1^										
	N1	N2					N3				
Ingredients ^2^, %		PC	LP	LPV	LPI	LPVI	PC	LP	LPV	LPI	LPVI
Corn, yellow dent	37.80	46.39	64.68	64.59	64.57	64.47	60.60	79.50	79.41	79.40	79.28
Soybean meal, 47.5% CP	16.25	26.64	8.50	8.50	8.50	8.50	23.33	5.00	5.00	5.00	5.00
Fish meal, menhaden	6.40	5.00	5.00	5.00	5.00	5.00	5.00	5.00	5.00	5.00	5.00
Whey, dried	24.50	4.50	4.50	4.50	4.50	4.50	—	—	—	—	—
Corn starch	—	14.00	12.30	12.30	12.30	12.30	8.00	5.90	5.90	5.90	5.90
Lactose	7.00	—	—	—	—	—	—	—	—	—	—
Plasma spray-dried	5.75	—	—	—	—	—	—	—	—	—	—
Corn oil	0.50	—	—	—	—	—	—	—	—	—	—
Dicalcium phosphate 18.5%	0.79	1.20	1.53	1.53	1.53	1.53	1.00	1.34	1.34	1.34	1.34
Limestone	0.39	0.38	0.30	0.30	0.30	0.30	0.33	0.23	0.23	0.23	0.23
Salt	0.12	0.67	0.68	0.68	0.68	0.68	0.58	0.58	0.58	0.58	0.58
Chromium oxide	—	0.50	0.50	0.50	0.50	0.50	0.50	0.50	0.50	0.50	0.50
Vitamin Premix	0.03	0.03	0.03	0.03	0.03	0.03	0.03	0.03	0.03	0.03	0.03
Trace Mineral Premix	—	0.01	0.02	0.02	0.02	0.02	0.01	0.01	0.01	0.01	0.01
Zinc oxide, 72% Zn	0.01	0.01	0.01	0.01	0.01	0.01	0.01	0.01	0.01	0.01	0.01
L-Lysine, HCl	0.27	0.41	0.87	0.87	0.87	0.87	0.39	0.85	0.85	0.85	0.85
DL-methionine	0.13	0.1	0.18	0.18	0.18	0.18	0.08	0.16	0.16	0.16	0.16
L-threonine	0.05	0.14	0.39	0.39	0.39	0.39	0.13	0.38	0.38	0.38	0.38
L-tryptophan	0.01	0.02	0.11	0.11	0.11	0.11	0.02	0.11	0.11	0.11	0.11
L-isoleucine	—	—	—	—	0.30	0.30	—	—	—	0.31	0.31
L-valine	—	—	—	0.31	—	0.31	—	—	0.31	—	0.31
L-alanine	—	—	0.40	0.18	0.21	—	—	0.40	0.18	0.19	—
Calculated Composition ^3^											
Dry matter, %	90.38	90.35	90.30	90.31	90.31	90.32	89.38	89.27	89.29	89.29	89.3
ME, Mcal/kg	3.41	3.40	3.40	3.40	3.40	3.40	3.36	3.36	3.36	3.36	3.36
Crude protein, %	21.90	20.00	14.00	14.00	14.00	14.00	19.00	13.00	13.00	13.00	13.00
Crude fiber, %	1.60	2.00	1.87	1.87	1.86	1.86	2.42	2.05	2.05	2.05	2.05
Crude fat, %	3.40	3.00	3.15	3.14	3.14	3.14	3.25	3.55	3.55	3.55	3.54
Calcium, %	0.85	0.80	0.80	0.80	0.80	0.80	0.70	0.70	0.70	0.70	0.70
Total phosphorus, %	0.70	0.65	0.65	0.65	0.65	0.65	0.60	0.60	0.60	0.60	0.60
Available phosphorus, %	0.62	0.46	0.49	0.49	0.49	0.49	0.39	0.43	0.43	0.43	0.43
Potassium, %	0.95	0.81	0.50	0.50	0.50	0.50	0.71	0.39	0.39	0.39	0.39
SID Lysine, %	1.50	1.35	1.35	1.35	1.35	1.35	1.23	1.23	1.23	1.23	1.23
SID Threonine, %	0.88	0.79	0.79	0.79	0.79	0.79	0.73	0.73	0.73	0.73	0.73
SID Methionine, %	0.43	0.39	0.39	0.39	0.39	0.39	0.36	0.36	0.36	0.36	0.36
SID Tryptophan, %	0.25	0.22	0.22	0.22	0.22	0.22	0.20	0.20	0.20	0.20	0.20
SID Isoleucine, %	0.78	0.73	0.43	0.43	0.73	0.73	0.68	0.37	0.37	0.68	0.68
SID Valine, %	0.96	0.81	0.50	0.81	0.50	0.81	0.77	0.46	0.77	0.46	0.77
SID Leucine, %	1.64	1.43	1.01	1.01	1.01	1.01	1.40	0.98	0.98	0.98	0.98
SID Histidine, %	0.50	0.45	0.28	0.28	0.28	0.28	0.43	0.27	0.27	0.27	0.27
SID Arginine, %	1.12	1.17	0.65	0.65	0.65	0.65	1.10	0.58	0.58	0.58	0.58
SID Phenylalanine, %	0.89	0.82	0.49	0.49	0.49	0.49	0.78	0.45	0.45	0.45	0.45
SID Phenylalanine + Tyrosine, %	1.64	1.46	0.89	0.89	0.89	0.89	1.39	0.82	0.82	0.82	0.82
SID Valine: SID Lysine	0.64	0.60	0.37	0.60	0.37	0.60	0.62	0.37	0.62	0.37	0.62
SID Isoleucine: SID Lysine	0.52	0.54	0.32	0.32	0.54	0.54	0.55	0.30	0.30	0.55	0.55

^1^PC (positive control): standard-protein diet; LP (negative control): low-protein diet containing limiting amino acids (i.e., Lys, Met, Thr and Trp) at NRC (2012) levels; LPV: LP containing Val at NRC level; LPI: LP containing Ile at NRC level; LPVI: LP containing Val and Ile at NRC levels. N1 diet was provided from days (d) 1 to 7 of the study. N2 diets were offered from d 8 to 21 of the study. N3 diets were provided from d 22 to 42 of the study. ^2^ Corn, soybean meal, fish meal, whey, corn starch, lactose, plasma spray-dried, corn oil, dicalcium phosphate, limestone, zinc oxide, salt, and lysine HCl (79–99%) were obtained from Nutra Blend, LLC (Neosho, MO). DL-methionine (99%) was obtained from Evonik (Kennesaw, GA, USA). L-threonine (98.5%) and L-tryptophan (98%) were obtained from Ajinomoto (Overland Park, KS, USA). L-isoleucine (98.5%), L-alanine, and L-valine (96.5%) were obtained from Ajinomoto Health & Nutrition North America, Inc. (Raleigh, NC, USA). Chromium oxide was purchased from Fisher Scientific (Bartlesville, OK, USA). Vitamin premix was purchased from Nutra Blend, LLC (Neosho, MO, USA) containing vitamin A, 1,650,000 IU/kg; vitamin D3, 660,000 IU/kg; vitamin E, 17,600 IU/kg; vitamin K (menadione), 1320 mg/kg; vitamin B12, 13.2 mg/kg; niacin, 19,800 mg/kg; D-pantothenic acid, 11,000 mg/kg; riboflavin, 3300 mg/kg; and phytase, 300,000 FYT/kg. Trace mineral premix was purchased from Nutra Blend, LLC (Neosho, MO, USA) containing copper, 11,000 ppm; iodine, 198 ppm; iron, 73,000 ppm; manganese, 22,000 ppm; selenium, 198 ppm; and zinc, 73,000 ppm. ^3^ Values were obtained using National Swine Nutrition Guide (NSNG; Version 2.1 Metric, ©2012 U.S. Pork Center of Excellence). SID: standardized ileal digestibility; ME: metabolizable energy.

**Table 5:** Analyzed chemical composition and amino acid content of experimental diets (as-fed basis).

	Diets ^1^										
	N1	N2					N3				
		PC	LP	LPV	LPI	LPVI	PC	LP	LPV	LPI	LPVI
Analyzed Composition, %											
Dry matter	89.70	88.30	88.50	88.90	87.70	89.80	87.80	88.20	87.90	87.5	88.30
Crude protein	20.94	18.44	13.80	14.28	13.81	13.50	18.74	12.31	12.90	12.90	12.60
Crude fiber	1.60	2.20	1.80	1.60	1.70	1.80	2.30	1.90	2.10	1.90	1.90
Calcium	1.03	0.92	0.76	0.97	0.90	0.93	0.83	0.76	0.71	0.71	0.79
Phosphorus	0.79	0.65	0.63	0.72	0.68	0.73	0.61	0.65	0.59	0.59	0.67
Isoleucine	0.91	0.79	0.52	0.54	0.77	0.79	0.79	0.42	0.45	0.72	0.78
Valine	1.14	0.88	0.60	1.02	0.58	0.90	0.88	0.50	0.89	0.54	0.88
Leucine	1.79	1.47	1.10	1.10	1.03	1.07	1.49	0.99	1.03	1.02	1.05
Lysine	1.65	1.43	1.39	1.48	1.46	1.42	1.43	1.23	1.32	1.35	1.33
Threonine	0.99	0.79	0.79	0.90	0.84	0.87	0.77	0.71	0.71	0.77	0.71
Methionine	0.44	0.40	0.42	0.40	0.40	0.42	0.42	0.35	0.39	0.37	0.41
Tryptophan	0.30	0.24	0.24	0.25	0.24	0.24	0.23	0.22	0.20	0.20	0.21
Arginine	1.14	1.09	0.70	0.72	0.66	0.68	1.13	0.56	0.62	0.61	0.62
Histidine	0.52	0.45	0.31	0.32	0.30	0.31	0.46	0.27	0.29	0.28	0.29
Phenylalanine	0.97	0.86	0.58	0.59	0.55	0.56	0.88	0.48	0.53	0.53	0.53
Hydroxyproline	0.11	0.11	0.11	0.12	0.11	0.12	0.09	0.12	0.12	0.11	0.10
Aspartic acid	2.02	1.75	1.08	1.14	1.04	1.09	1.75	0.86	0.93	0.92	0.93
Serine	0.88	0.70	0.49	0.49	0.46	0.47	0.72	0.41	0.44	0.43	0.45
Glutamic acid	3.32	3.01	2.03	2.06	1.91	1.98	3.06	1.72	1.80	1.79	1.80
Proline	1.15	1.01	0.78	0.76	0.73	0.78	1.01	0.75	0.75	0.71	0.75
Glycine	0.91	0.85	0.61	0.65	0.59	0.62	0.88	0.55	0.58	0.58	0.57
Alanine	1.08	0.93	1.09	0.91	0.86	0.73	0.95	1.23	0.98	0.91	0.72
Cysteine	0.38	0.27	0.21	0.21	0.18	0.20	0.29	0.18	0.19	0.19	0.19
Tyrosine	0.67	0.57	0.40	0.41	0.37	0.38	0.58	0.33	0.37	0.37	0.38
Hydroxylysine	0.04	0.04	0.04	0.04	0.04	0.04	0.05	0.03	0.04	0.04	0.04
Taurine ^2^	0.20	0.20	0.20	0.20	0.21	0.21	0.20	0.21	0.21	0.22	0.20
Lanthionine ^2^	0.02	0.01	0.00	0.00	0.00	0.00	0.01	0.00	0.00	0.00	0.00
Ornithine ^2^	0.02	0.02	0.01	0.01	0.01	0.01	0.02	0.01	0.01	0.01	0.01
Valine: Lysine	0.69	0.61	0.43	0.68	0.40	0.63	0.61	0.40	0.67	0.40	0.66
Isoleucine: Lysine	0.55	0.55	0.37	0.36	0.53	0.55	0.55	0.34	0.34	0.54	0.58

^1^PC (positive control): standard-protein diet; LP (negative control): low-protein diet containing limiting amino acids (i.e., Lys, Met, Thr and Trp) at NRC (2012) levels; LPV: LP containing Val at NRC level; LPI: LP containing Ile at NRC level; LPVI: LP containing Val and Ile at NRC levels. N1 diet was provided from days (d) 1 to 7 of the study. N2 diets were offered from d 8 to 21 of the study. N3 diets were provided from d 22 to 42 of the study. ^2^ Non-proteinogenic amino acids.

## Data Availability

Datasets supporting the results of this article are included within the article and its supplementary files.

## Supplementary Materials

The following supporting information can be downloaded at: https://www.mdpi.com/article/10.3390/ijms23063300/s1.
