# Supplementary material for: A Mixture of Valine and Isoleucine Restores the Growth of Protein-Restricted Pigs Likely through Improved Gut Development, Hepatic IGF-1 Pathway, and Plasma Metabolomic Profile
Source: Int J Mol Sci. 2022 Mar 18;23(6):3300. doi: 10.3390/ijms23063300 (PMC8955368; doi:10.3390/ijms23063300)
Supplement: Supplementary file 1 [file ijms-23-03300-s001.zip › ijms-1628056-supplementary.pdf]

Supplementary Material

Supplementary Tables

Table S1. Weekly growth measurements of nursery pigs fed with very low protein diets supplemented with Ile, Val, or combination of both

	Diets[1]						
	PC	LP	LPV	LPI	LPVI	SEM[2]	*P*-value
**BWG[1], kg**							
Wk 1	2.00^{a}	1.14^{bc}	1.84$^{ab\Psi}$	0.94^{c}	1.80^{ab}	0.10	< 0.01
Wk 2	2.86^{a}	1.97^{ab§}	2.46^{a}	1.26^{b}	2.38^{a}	0.13	< 0.01
Wk 3	3.46^{a}	1.73^{bc}	1.80^{bc}	1.21^{c}	2.51$^{ab\#}$	0.17	< 0.01
Wk 4	4.16^{a}	2.14^{cd}	3.33^{ab}	1.47^{d}	3.16^{abc}	0.19	< 0.01
Wk 5	4.50^{a}	2.72^{b}	3.46^{ab}	2.31^{b}	4.21^{a}	0.19	< 0.01
**MFI[1], kg**							
Wk 1	0.33^{ab}	0.33^{ab}	0.37^{a}	0.26^{b}	0.40^{a}	0.01	< 0.01
Wk 2	0.56^{abc}	0.46^{bc}	0.59^{ab}	0.41$^{c¥}$	0.65^{a}	0.02	< 0.01
Wk 3	0.75^{ab}	0.60^{bc}	0.65^{ab}	0.45^{c}	0.79$^{ab\&}$	0.03	< 0.01
Wk 4	0.95^{a}	0.73^{bc}	0.82^{ab}	0.54$^{c\$}$	0.91$^{ab\&}$	0.03	< 0.01
Wk 5	1.12^{a}	0.81^{bc}	0.97^{ab}	0.63^{c}	1.18^{a}	0.04	< 0.01
**CFI [1], kg**							
Wk1	2.27^{ab}	2.31^{ab}	2.59^{a}	1.84^{b}	2.78^{a}	0.09	< 0.01

Wk 2	3.91^{abc}	3.20^{bc}	4.12^{ab}	2.86$^{c¥}$	4.55^{a}	0.15	< 0.01
Wk 3	5.28^{ab}	4.19^{abc}	4.53^{ab}	3.11^{c}	5.52$^{a\&}$	0.20	< 0.01
Wk 4	6.68^{a}	5.09^{bc}	5.74^{ab}	3.81$^{c\$}$	6.38$^{ab\&}$	0.22	< 0.01
Wk 5	7.84^{a}	5.69^{bc}	6.79^{ab}	4.42^{c}	8.25^{a}	0.32	< 0.01
**CPI [1], kg**							
Wk 1	0.43^{a}	0.30^{bc}	0.35^{ab}	0.24^{c}	0.41^{a}	0.02	< 0.01
Wk 2	0.73^{a}	0.44b^{c}	0.59^{ab}	0.39^{c}	0.63^{a}	0.03	< 0.01
Wk 3	0.99^{a}	0.49^{bc}	0.59^{bc}	0.40^{c‡}	0.69$^{b\&}$	0.04	< 0.01
Wk 4	1.25^{a}	0.62^{bc}	0.75^{b}	0.49^{c}	0.80$^{b\&}$	0.05	< 0.01
Wk 5	1.47^{a}	0.69^{cd}	0.88^{bc}	0.57^{d}	1.04^{b}	0.06	< 0.01
**G:F [1], kg/kg**							
Wk 1	0.92^{a}	0.53^{b}	0.70^{ab}	0.58^{b}	0.66^{b}	0.03	< 0.01
Wk 2	0.79^{a}	0.58^{ab§}	0.59^{ab*}	0.45^{b}	0.53^{b}	0.03	< 0.01
Wk 3	0.81	0.54	0.41*	0.37$^{¥}$	0.46	0.05	0.04
Wk 4	0.71^{a}	0.41^{b}	0.57^{ab}	0.38^{b}	0.49^{b}	0.03	< 0.01
Wk 5	0.67	0.55	0.51	0.50	0.51	0.03	0.52
**G:P [1], kg/kg**							
Wk 1	4.94	4.03	4.91	4.17	4.70	0.20	0.51
Wk 2	3.86	3.93	4.15	3.27	3.74	0.15	0.35
Wk 3	3.67	3.07	3.15	2.89	3.60	0.13	0.24
Wk 4	3.29^{b}	3.14^{b}	4.40^{a}	2.97^{b}	3.90$^{ab@}$	0.13	< 0.01
Wk 5	3.09^{b}	3.37^{ab}	3.73^{ab}	3.91$^{ab¥}$	4.08^{a}	0.11	0.01

[1]PC (positive control), standard protein diet; LP (negative control), low protein diet containing limiting amino acids (i.e., Lys, Met, Thr and Trp) at NRC (2012) levels; LPV, LP containing Val at NRC level; LPI: LP containing Ile at NRC level; LPVI: LP containing Val and Ile at NRC levels. BWG: body weight gain; MFI: mean feed intake; CFI: cumulative feed intake; CPI: cumulative protein intake; G:F: gain:feed ratio; G:P: gain:protein ratio. The values are means, n=8. The P-values for the overall model effect for diet, week (wk) and diet × wk for BWG, MFI, CFI and CPI were < 0.01, < 0.01 and < 0.01, for G:F were < 0.01, 0.02 and 0.19 and for G:P were < 0.01, < 0.01 and 0.32, respectively.

[2]SEM: standard error of the mean.

[a,b,c,d] Within each row, the values with different superscript letter(s) are different ($P \leq 0.05$). [§] $P \leq 0.1$ LP vs. PC, [*] $P \leq 0.1$ LPV vs. PC, [¥] $P \leq 0.1$ LPI vs. PC, [#] $P \leq 0.1$ LPVI vs. PC, [Ψ] $P \leq 0.1$ LPV vs. LP, [$] $P \leq 0.1$ LPI vs. LP, [&] $P \leq 0.1$ LPVI vs. LP, [‡] $P \leq 0.1$ LPI vs. LPV, [@] $P \leq 0.1$ LPVI vs. LPI.

Table S2. Non-significant plasma metabolites in nursery pigs fed with very low protein diets supplemented with Ile, Val, or combination of both.

**Metabolites**	**Diets[1]**						
	**PC**	**LP**	**LPV**	**LPI**	**LPVI**	**SEM[2]**	***P*-value**
Pelargonic acid	16743	16562	16456	16527	16528	82	0.86
Phenylacetic acid	4037	3473	3340	3865	2898	175	0.26
Phenylethylamine	1759	1742	1685	1744	1769	34	0.95
Phosphate	486833	449282	388919	357830	404195	18840	0.21
Phosphoethanolamine	10374	9787	10056	9985	9970	93	0.42
Phytosphingosine	1272	901	1220	999	1391	69	0.14
Pipecolinic acid	17218	19063	18679	19960	19468	431	0.32
Proline	1106940	1189833	1139922	1170529	1144801	10379	0.10
Pseudo uridine	24544	24294	24579	24397	24786	174	0.93
Putrescine	5063	4855	5101	4792	4719	183	0.96
Ribitol	4322	4052	5404	4103	4317	257	0.47

Ribonic acid	65535	46953	61598	48444	47370	3414	0.25
Ribose	19431	14923	14612	16988	16650	985	0.57
Salicylic acid	1885	1924	1955	1949	2106	29	0.19
Sophorose	807	982	1055	956	1172	71	0.60
Stearic acid	761930	756035	749177	738208	761551	4805	0.51
Succinic acid	81651	81010	77006	79173	77448	1052	0.57
Taurine	5989	6991	7181	6909	6420	247	0.56
Threitol	7104	7317	7123	7168	7264	46	0.55
Thymidine	3513	3472	3485	3549	3514	27	0.91
Thymine	2023	1795	1675	2008	1954	94	0.74
Trans 4 hydroxyproline	395255	346104	368271	384076	401834	10245	0.45
Tryptophan	237901	315808	326614	278326	307687	11671	0.10
UDP glucuronic acid	1689	1568	1604	1554	1521	46	0.83
Uric acid	1870	1809	1800	1822	1754	20	0.48
Uridine	2064	1908	2012	1982	2000	27	0.51

Xanthine	1279	1177	1225	1160	1205	17	0.19
Xylose	17373	17737	17038	17505	17034	107	0.17
1,5-anhydroglucitol	129894	129333	123970	115899	157050	6577	0.36
1-methylhydantoin	11388	12221	11148	11706	11762	140	0.15
2-hydroxybutanoic acid	53352	53578	53172	54825	53087	397	0.64
2-picolinic acid	684	675	710	692	691	10	0.90
3,4-dihydroxycinnamic acid	655	657	670	651	666	6	0.86
4-hydroxyhippuric acid	413	388	409	421	374	20	0.95
4-hydroxyphenylacetic acid	1003	1001	972	1027	1038	11	0.38
5-methoxytryptamine	1096	1051	1119	1176	982	44	0.72
Aconitic acid	2565	2526	2459	2437	2486	20	0.28
Adenine	1280	1232	1238	1191	1228	11	0.18
Adenosine-5-monophosphate	551	553	602	564	568	15	0.87
Adipic acid	3913	3895	3842	3829	3917	31	0.86
Allantoic acid	27984	26824	27034	27356	27816	226	0.44
Anthranilic acid	823	790	824	788	837	8	0.13

Arachidic acid	5117	4868	4917	4765	5007	49	0.18
Arachidonic acid	19440	22939	25626	23166	25521	1409	0.65
Asparagine	37597	37527	36851	36811	36360	206	0.28
Aspartic acid	54981	53509	55281	52802	55484	386	0.11
β-glycerolphosphate	3606	5486	3886	4154	4830	255	0.12
β-mannosylglycerate	2674	2753	3507	2682	4203	263	0.25
β-sitosterol	1545	1591	1610	1534	1591	16	0.54
Biphenyl	1746	1903	1895	1882	1883	25	0.22
Cadaverine	2908	2806	2834	2822	2906	20	0.31
Capric acid	3480	3426	3725	3213	3663	116	0.68
Caprylic acid	11837	11373	11608	11575	11600	67	0.34
Cerotinic acid	789	784	758	751	792	8	0.39
Chenodeoxycholic acid	5622	5490	5520	5605	5575	70	0.98
Cholesterol	298448	314443	299453	308406	357306	11180	0.46
Citric acid	354835	340240	345232	337763	344527	2296	0.16

Citrulline	45150	35027	36322	35637	38642	1557	0.23
Cyano-L-Alanine	2440	3124	2851	2941	2661	143	0.63
Cysteine	4245	4401	4401	4590	4435	50	0.30
Cytosine	1307	1275	1285	1248	1276	7	0.17
Dihydroxymalonic acid	578	453	678	579	513	41	0.52
Dimethylglycine	1476	3409	2662	3040	2699	238	0.11
Docosahexaenoic acid	3991	4166	3942	4096	4131	41	0.37
Erythritol	34556	35338	34825	34720	35472	185	0.45
Erythrose	973	971	979	994	993	9	0.89
Fumaric acid	10438	10307	9893	10204	9689	107	0.16
Galactinol	602	571	609	581	574	6	0.11
Glucose-6-phosphate	7738	7002	7095	6734	7193	127	0.14
Glucuronic acid	17927	16804	17400	17545	17245	144	0.18
Glycerol	212290	202758	201154	195734	197990	2334	0.23
Glycerol-alpha-phosphate	49416	44721	42679	47746	41094	1961	0.67
Glycyl tyrosine	775	840	803	771	761	12	0.26

Guanidinosuccinate	1405	1251	1542	1273	1351	56	0.51
Guanosine	2485	2212	2312	2251	2341	41	0.25
Heptadecanoic acid	53327	68765	60100	63311	59173	2930	0.58
Hypoxanthine	148884	126372	133377	107891	135201	7549	0.55
Indol-3-propionic acid	8692	8757	8179	8654	8530	121	0.59
Indol-3-lactate	9037	8659	8608	8578	8746	79	0.35
Inosine-5-monophosphate	438	417	419	414	413	4	0.38
Inosine	23355	21557	22204	20772	21827	458	0.49
Inositol-4-monophosphate	947	956	848	947	933	62	0.98
Isocitric acid	7145	6913	6997	6853	6977	44	0.28
Isomaltose	2040	2100	2044	2113	2088	27	0.89
Isothreonic acid	73413	60222	72687	57376	62540	2616	0.17
Lactamide	4145	3983	3979	3916	3860	34	0.11
Lactic acid	3970671	4107989	3609044	3161065	3348051	198315	0.53
Lactose	1719	1643	1650	1576	1646	19	0.24

Linoleic acid	6862	8027	6254	8551	6499	409	0.32
Maleimide	4419	4391	4215	4623	4367	132	0.92
Maltitol	443	428	437	416	429	6	0.67
Methanolphosphate	4194	5239	4913	5914	5918	253	0.15
Myo-inositol	354815	356919	344019	355843	353453	3378	0.77
Myristic acid	4818	3891	3936	3691	3534	172	0.15
N-acetyl-aspartic acid	1883	1862	1785	1862	1847	31	0.91
N-acetylglycine	1620	1430	1614	1780	1755	93	0.79
N-acetylmannosamine	2112	1915	2076	1846	2333	111	0.69
Nicotinic acid	858	855	846	832	833	7	0.66
Oleic acid	9377	9699	7660	9195	7729	632	0.78
Oxalic acid	3132	3201	3196	3176	3157	42	0.99
Palmitic acid	161805	143024	135382	128512	143851	5356	0.37
Palmitoleic acid	689	674	684	682	693	6	0.89
Pantothenic acid	863	672	762	704	863	46	0.59

[1]PC (positive control), standard protein diet; LP (negative control), low protein diet containing limiting amino acids (i.e., Lys, Met, Thr and Trp) at NRC (2012) levels; LPV, LP containing Val at NRC level; LPI: LP containing Ile at NRC level; LPVI: LP containing Val and Ile at NRC levels. The values are mean peak heights, *n*=8.

[2]SEM: standard error of the mean.

Table S3. The sequences [forward (F) and reveres (R)], location on template, amplicon size (bp), and GenBank accession numbers for primers used for reverse transcription quantitative real-time polymerase chain reaction (RT-qPCR)

**Genes**[1]	**Sequence (5' → 3')**	**Location on template**	**Amplicon length (bp)**	**GenBank accession no.**
MUC1	**F:** GTGCCGCTGCCCACAACCTG	333-352	141	XM_021089730.1
	**R:** AGCCGGGTACCCCAGACCCA	454-473		
MUC2	**F:** GGTCATGCTGGAGCTGGACAGT	483-504	181	XM_021082584.1
	**R:** TGCCTCCTCGGGGTCGTCAC	644-663		
EGFR	**F:** GCCTTAGCCGTCTTATCCAA	478-497	299	NM_214007.1
	**R:** TGGGCACAGATGACTTTGGT	757-776		
IGF-1R	**F:** TTCGCCAGATCCTAGGGGAG	1242-1261	120	NM_214172.1
	**R:** TCCCAGCTTTGATGGTCAGG	1342-1361		
Caspase-9	**F:** AATGCCGATTTGGCTTACGT	606-625	195	XM_013998997.2
	**R:** CATTTGCTTGGCAGTCAGGTT	780-800		
Ki-67	**F:** TCTTGTCCCTGAATCCGCAA	86-105	88	NM_001101827.1
	**R:** TGTTTCTCTGGTTGCTTGGTTG	152-173		

PPARγ	**F:** AGGACTACCAAAGTGCCATCAAA	312-334	142	NM_214379.1
	**R:** GAGGCTTTATCCCCACAGACAC	432-453		
IGF-1	**F:** CACAGACGGGCATCGTGGAT	383-402	90	XM_021091138.1
	**R:** ACTTGGCAGGCTTGAGGGGT	453-472		
IGFBP1	**F:** CCCATCCTTTGGAACGCCATCAAT	467-490	121	NM_001195105.1
	**R:** TGGCTAGTCTGTCCAGCACTTTGT	564-587		
mTOR	**F:** GGGGTTTGGATCAGGGTCTG	2504-2523	80	XM_003127584.6
	**R:** GACTCATCCGCCCCTACATG	2444-2463		
GCN2	**F:** GAAATGCTGGAAAGGCAGGC	526-545	77	XM_021097873.1
	**R:** CGTTGCTCCTGCTCCTCTTT	583-602		
ATF4	**F:** GGGCTGAAGAGAGCTTAGGG	1068-1087	69	XM_021090887.1
	**R:** ACCCATGAGGTTTGAAGTGC	1117-1136		
CSNK2A1	**F:** TTTACCTCCAGCTGCCTCTT	191-210	151	XM_003134359.5
	**R:** AGTACTCTCGGGGTCTGTGC	322-341		
PRKCA	**F:** GAAGGGCAGTTTCGGGAAGG	1038-1057	156	XM_021066740.1
	**R:** GGAGGCTTATCCAACAGGGC	1174-1193		

OCLN	**F:** TCCTGGGTGTGATGGTGTTC	633-652	144	NM_001163647.2
	**R:** CGTAGAGTCCAGTCACCGCA	757-776		
ZO1	**F:** AAGCCCTAAGTTCAATCACAATCT	4981-5004	130	XM_021098827.1
	**R:** ATCAAACTCAGGAGGCGGC	5092-5110		
β-Actin	**F:** CTGCGGCATCCACGAAACT	944-962	147	XM_021086047.1
	**R:** AGGGCCGTGATCTCCTTCTG	1071-1090		

[1] *MUC1*, mucin 1; *MUC2*, mucin 2; *EGFR*, epidermal growth factor receptor; *IGF-1R*, insulin-like growth factor 1 receptor; *Ki-67; PPARγ,* peroxisome proliferator activated receptor gamma; *IGF-1*, insulin-like growth factor 1; *IGFBP1*, insulin-like growth factor binding protein 1; *mTOR*, mechanistic target of rapamycin; *GCN*2, general control nonderepressible 2; *ATF4*, activating transcription factor 4; *CSNK2A1*, casein kinase 2 alpha 1; *PRKCA*, protein kinase C alpha; *OCLN,* occludin; *ZO1*, zonula occludens 1.

Supplementary Figures

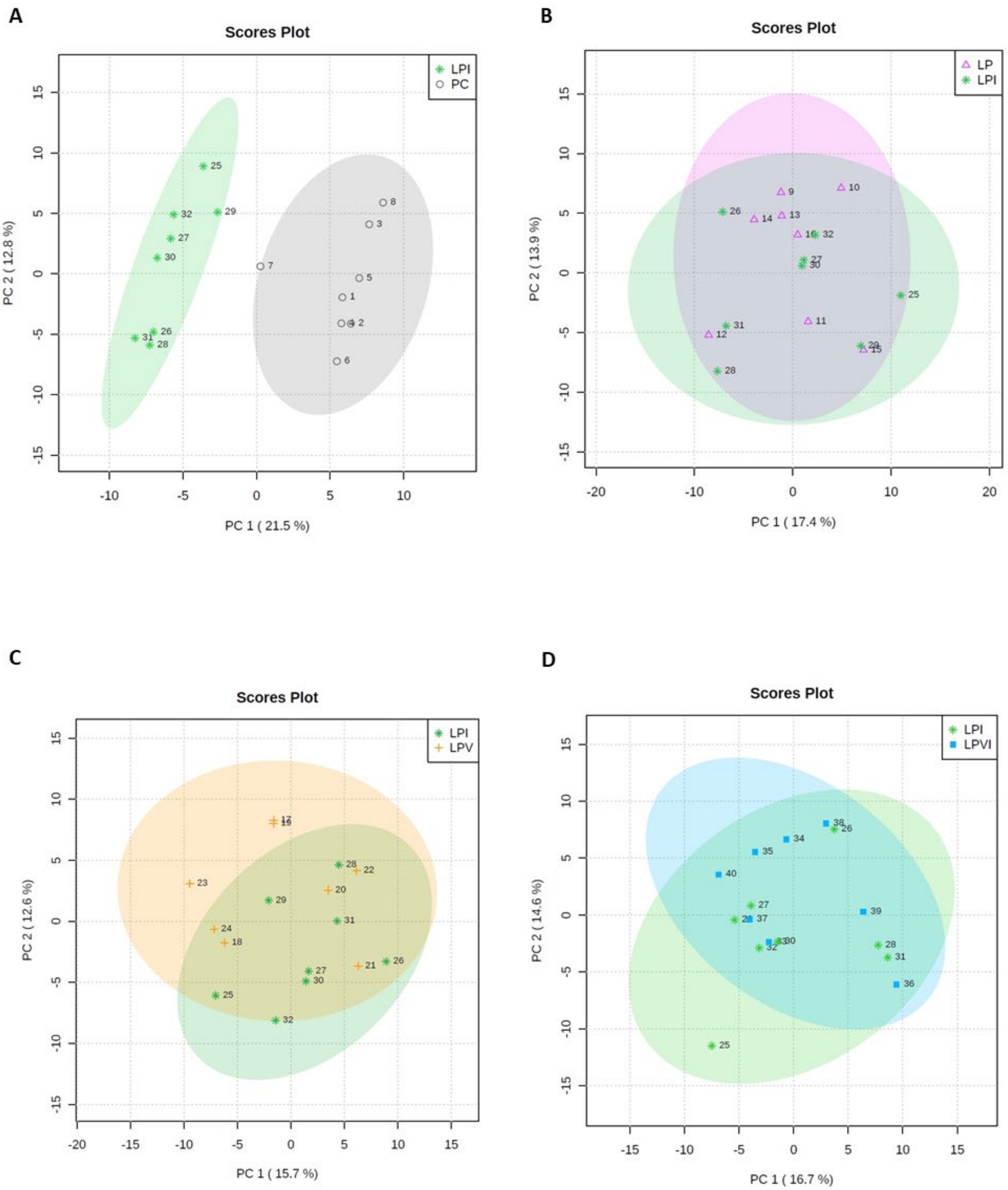

vs LP, (**C**) LPI vs LPV, (**D**) LPVI vs LPI. PC (positive control), standard protein diet; LP (negative control), low protein diet containing limiting amino acids (i.e., Lys, Met, Thr and Trp) at NRC (2012) levels; LPV, LP containing Val at NRC level; LPI: LP containing Ile at NRC level; LPVI: LP containing Val and Ile at NRC levels. Each shape represents a pig. *n*= 8.

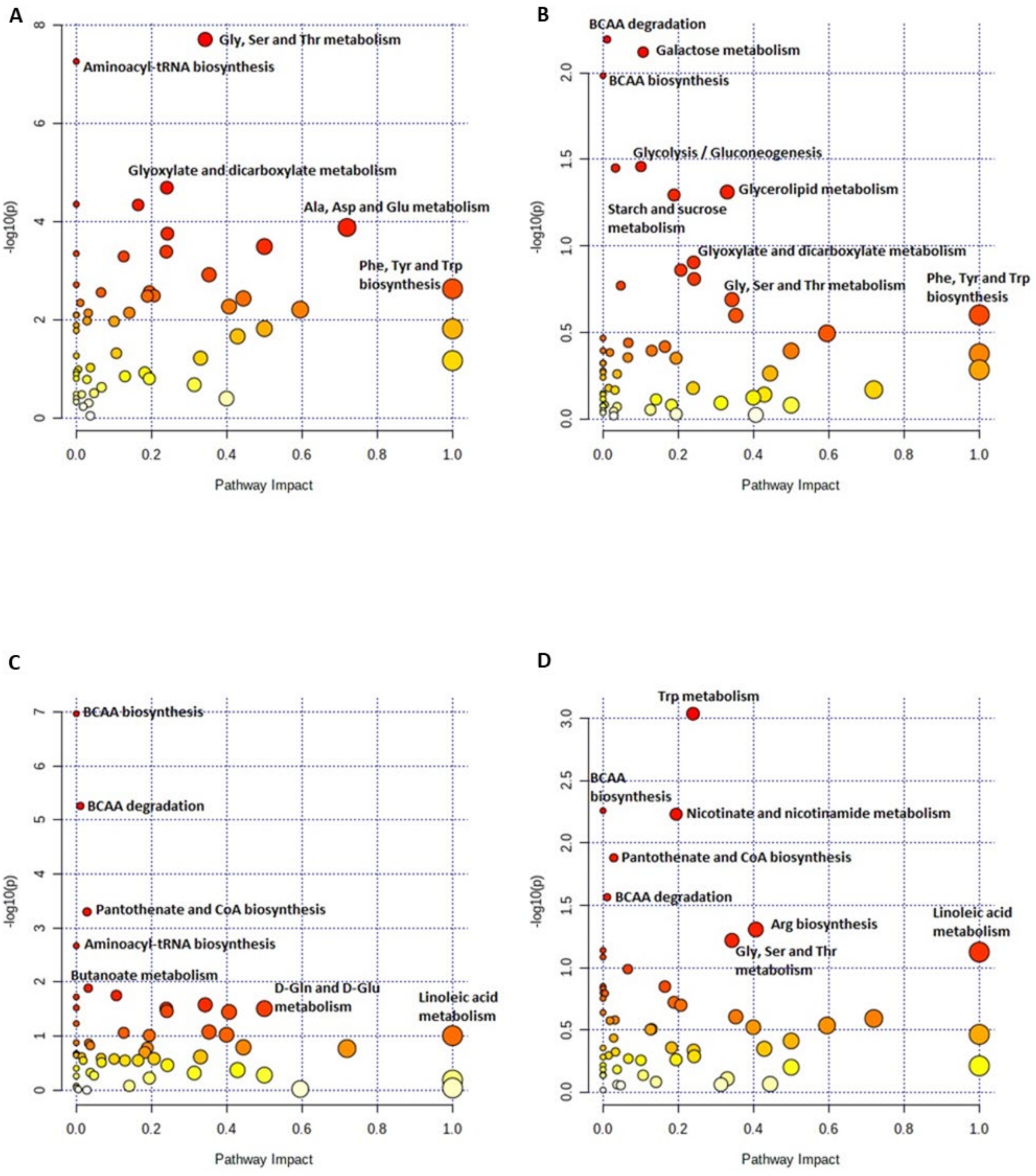

Figure S2. Pathway analysis map of plasma metabolites in nursery pigs fed with very low protein diets supplemented with Ile, Val, or combination of both. (**A**) LPI vs PC, (**B**) LPI vs LP, (**C**) LPI vs LPV, (**D**) LPVI vs LPI. PC (positive control), standard protein diet; LP (negative control), low protein diet containing limiting amino acids (i.e., Lys, Met, Thr and Trp) at NRC (2012) levels; LPV, LP containing Val at NRC

level; LPI: LP containing Ile at NRC level; LPVI: LP containing Val and Ile at NRC levels. Each circle is obtained from topology analysis representing a metabolic pathway with the scores. The x-axis indicates the pathway impact, and the y-axis shows the pathway enrichment. The circle size depends on its impact value while its color is based on its *P* value (i.e., greater circle size shows higher pathway impact, while darker color circles demonstrate more significant changes of metabolites and pathway enrichment). *n*= 8.

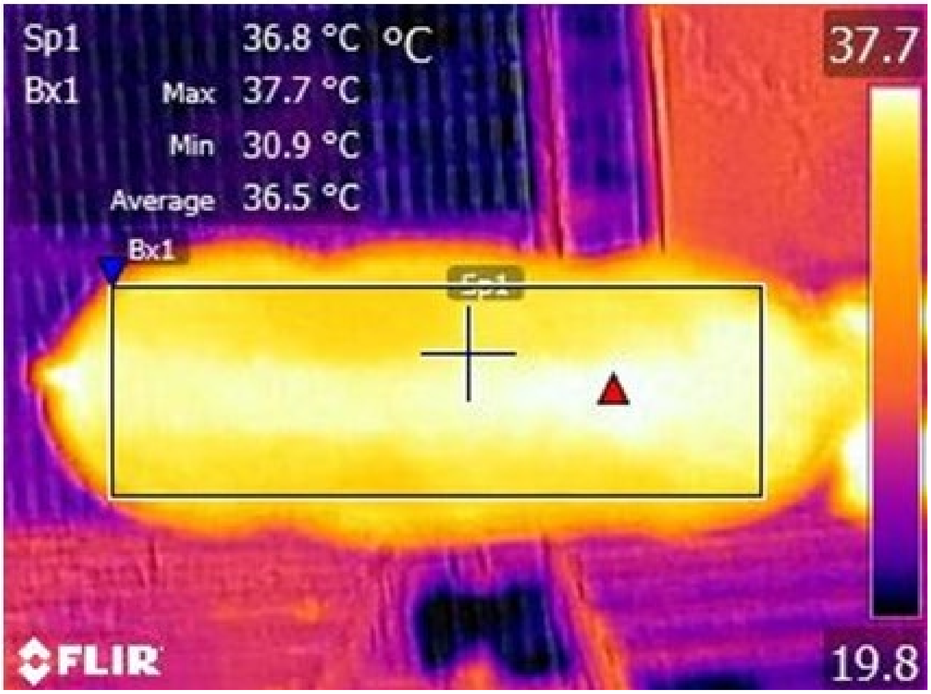

Figure S3. A representative picture of a thermal image. A FLIR camera software (FLIR Research Studio, Wilsonville, OR) was used to obtain the mean dorsal body surface temperature via drawing a rectangular region of interest in the back of pigs from shoulder to rump.
